# DNA methylation maintains the CLDN1-EPHB6-SLUG axis to enhance chemotherapeutic efficacy and inhibit lung cancer progression

**DOI:** 10.7150/thno.45785

**Published:** 2020-07-11

**Authors:** Jia-En Wu, Yi-Ying Wu, Chia-Hao Tung, Yao-Tsung Tsai, Hsuan-Yu Chen, Yuh-Ling Chen, Tse-Ming Hong

**Affiliations:** 1 Institute of Basic Medical Sciences, College of Medicine, National Cheng Kung University, Tainan, Taiwan.; 2 Clinical Medicine Research Center, National Cheng Kung University Hospital, College of Medicine, National Cheng Kung University, Tainan, Taiwan.; 3 Institute of Statistical Science, Academia Sinica, Taipei, Taiwan.; 4 Institute of Oral Medicine, College of Medicine, National Cheng Kung University, Tainan, Taiwan.; 5 Institute of Clinical Medicine, College of Medicine, National Cheng Kung University, Tainan, Taiwan.

**Keywords:** DNA methylation, CLDN1, cancer stem-like cells, drug resistance, metastasis.

## Abstract

The loss of cancer-cell junctions and escape from the primary-tumor microenvironment are hallmarks of metastasis. A tight-junction protein, Claudin 1 (CLDN1), is a metastasis suppressor in lung adenocarcinoma. However, as a metastasis suppressor, the underlying molecular mechanisms of CLDN1 has not been well studied.

**Methods:** The signaling pathway regulated by CLDN1 was analyzed by Metacore software and validated by immunoblots. The effect of the CLDN1-EPHB6-ERK-SLUG axis on the formation of cancer stem-like cells, drug resistance and metastasis were evaluated by sphere assay, aldefluor assay, flow cytometry, migration assay, cytotoxicity, soft agar assay, immunoprecipitation assay and xenograft experiments. Furthermore, the methylation-specific PCR, pyrosequencing assay, chromatin immunoprecipitation and reporter assay were used to study the epigenetic and RUNX3-mediated *CLDN1* transcription. Finally, the molecular signatures of RUNX3/CLDN1/SLUG were used to evaluate the correlation with overall survival by using gene expression omnibus (GEO) data.

**Results**: We demonstrated that CLDN1 repressed cancer progression via a feedback loop of the CLDN1-EPHB6-ERK1/2-SLUG axis, which repressed metastasis, drug resistance, and cancer stemness, indicating that CLDN1 acts as a metastasis suppressor. CLDN1 upregulated the cellular level of EPHB6 and enhanced its activation, resulting in suppression of ERK1/2 signaling. Interestingly, DNA hypermethylation of the *CLDN1* promoter abrogated SLUG-mediated suppression of* CLDN1* in low-metastatic cancer cells. In contrast, the histone deacetylase inhibitor trichostatin A or vorinostat facilitated *CLDN1* expression in high-metastatic cancer cells and thus increased the efficacy of chemotherapy. Combined treatment with cisplatin and trichostatin A or vorinostat had a synergistic effect on cancer-cell death.

**Conclusions:** This study revealed that DNA methylation maintains CLDN1 expression and then represses lung cancer progression via the CLDN1-EPHB6-ERK1/2-SLUG axis. Because CLDN1 enhances the efficacy of chemotherapy, CLDN1 is not only a prognostic marker but a predictive marker for lung adenocarcinoma patients who are good candidates for chemotherapy. Forced CLDN1 expression in low CLDN1-expressing lung adenocarcinoma will increase the chemotherapy response, providing a novel therapeutic strategy.

## Introduction

Metastasis is the major contributor to the high mortality of lung cancer. The escape of cancer cells from the primary-tumor microenvironment is the crucial point for metastasis 1, 2, and the loss of intercellular junctions is required for escape 3, 4. Therefore, tight junctions not only serve as a barrier to prevent leakage of molecules from the paracellular gap but also play an important role in repressing dissemination of cancer cells 5. For dissemination and subsequent colonization of a distant organ, the transformed epithelial cells often acquire a mesenchymal phenotype, a process termed the epithelial-mesenchymal transition (EMT). To drive EMT processes, EMT-specific regulators such as SLUG and SNAIL must be expressed and activated. These regulators also have been shown to facilitate cancer progression, including tumor-cell migration capacity and the generation of cancer stem-like cells (CSCs); they may also contribute to drug resistance 6-8 and be the potential therapeutic targets 9. During the EMT process, the epigenetic state of genes encoding these EMT regulators as well as epithelial cell-specific genes underlie the mechanism by which epithelial cells undergo EMT. Subsequently, epigenetics modulates cancer-cell plasticity 10, 11.

Epigenetic factors initially control the developmental program of embryogenesis 12. However, malignant tumors can hijack epigenetic mediators to promote cancer progression 13. During tumorigenesis, tumor-suppressor genes are often silenced by epigenetic regulation including DNA methylation and histone modifications. Owing to polycomb-mediated suppression, one type of histone modification is easily reversible, and the dynamics of this modification act in concert with DNA methylation to silence genes 14, 15. All polycomb target genes are subject to bivalent modification, i.e., with the repressive trimethyl-H3K4 (H3K27me3) mark and the activating trimethyl-H3K27 (H3K4me3) mark 16. Interestingly, the genes modulated by polycomb complexes frequently contain a CpG island in their promoters 15. The region of DNA methylation is always located in a CpG island and the methylation of CpG island often represents gene silence. Recently, the DNA methylation signature could correlate with chemotherapeutic drug efficiency, patient prognosis, and cancer metastasis. 17, 18.

Claudins are key components of tight junctions. They are transmembrane proteins that are anchored to actin filaments through ZO-1 and ZO-2 19. These proteins may mediate claudin signals that are transduced to the nucleus 20. Aberrant expression of claudin members has been reported in various cancers 21. Notably, claudin-1 (CLDN1) is downregulated in several cancers, and this correlates with recurrence or metastatic phenotype of lung adenocarcinoma, gastric cancer, breast cancer, melanoma, and colon cancer 22-26. Additionally, in tumors, the presence of subpopulations of breast-cancer cells in which claudin is downregulated correlates with a CSC phenotype and resistance to chemotherapy 27. Although the downregulation of CLDN1 is involved in cancer malignancy, how the expression of CLDN1 is controlled and the action mechanism of CLDN1 remain unclear.

The ephrin receptor (EPH) and its ligand constitute a bidirectional signaling pathway that contributes to the formation of subcellular compartments and to cell movement 28, 29. EPHB signaling modulates the function of adherens junctions and regulates cell migration in the intestinal epithelium 30. Notably, EPHB6 suppresses metastasis in non-small-cell lung cancer 31. It also inhibits breast-cancer invasiveness and, consequently, may prove useful for breast-cancer diagnosis and prognosis 32-34. Additionally, the EPHB collaborate with WNT signaling with respect to regulating cell migration and proliferation in the intestinal stem-cell niche 35. Furthermore, EPH/ephrin signaling can regulate MAPK signaling 36.

Here, we demonstrate that activation of the CLDN1-EPHB6-SLUG axis can repress lung-cancer progression and hence increase patient survival. Moreover, *CLDN1* expression was found to be driven by RUNX3 and epigenetically regulated by DNA methylation, which prevented SLUG binding to the* CLDN1* promoter and thus abrogated SLUG-mediated transcriptional repression of *CLDN1*. Finally, our results indicate that CLDN1 is both a prognostic marker and a predictive marker for the patients who respond well to chemotherapy.

## Materials and Methods

### Antibodies and cell lines

All antibodies used in this study are listed in Table S1. The human lung adenocarcinoma cell lines CL_1-0_, CL_1-5_ and Hop62 were maintained in RPMI 1640 medium supplemented with 10% fetal bovine serum (FBS, Gibco) and penicillin/streptomycin/antimycotic (Corning, USA). CL_1-5_ cell line was selected from the CL_1-0_ cell line by* in vitro* transwell selection. Hop62 cells (lung adenocarcinoma) originated from the Developmental Therapeutics Program of the National Cancer Institute (Bethesda, MD, USA). A549 (lung adenocarcinoma) and Hs68 (immortalized human fibroblast) cells originated from American Type Culture Collection and were cultured in Dulbecco's Modified Eagle Medium containing 10% fetal bovine serum (FBS, Gibco) and penicillin/streptomycin/antimycotic (Corning). The stable cell lines were maintained in the same medium used to culture the parental cells and selected using G418 (500 μg/mL) or puromycin (2 μg/mL), depending on the resistance marker encoded by the relevant individual plasmid. Cisplatin-resistant A549 cells were obtained from A549 cells treated with slowly increasing the concentration of cisplatin for six months in our laboratory. All cell lines were incubated at 37 °C in a humidified atmosphere containing 5% CO_2_.

### Reagents

The ephrin-B2 Fc was purchased from R&D Systems (7397-EB). Proteinase K was purchased from MERCK (1245680100). RNase A and DNase I were purchased from Sigma Aldrich (R4642 and D4527). N-2 Supplement was purchased from Invitrogen (17502048). Recombinant human epidermal growth factor and bovine fibroblast growth factor were purchased from PEPROTECH (100-18B and AF-100-15). The DNA methyltransferase inhibitor 5'Aza (1854), the HDAC inhibitors TSA (1606) and vorinostat (1604), and MEK1/2 inhibitors PD98059 (1666) were purchased from BioVision.

### Plasmid construction

The *CLDN1* cDNA was cloned into three plasmids, including pCI-neo plasmid by XhoI and NotI restriction enzyme, pcDNA3.1-HA-CPO plasmid by RsrII restriction enzyme, and pEGFP-C1 plasmid by XhoI and BamHI restriction enzyme. The *EPHB6* cDNA was cloned into pSec-Tag2 plasmid by BamHI and XhoI restriction enzyme. The *SLUG* cDNA was cloned into pCI-neo plasmid by EcoRI and SalI restriction enzyme. The *RUNX3* cDNA was cloned into pcDNA3.1-HA-CPO and pFlag-CMV2-CPO plasmids by RsrII restriction enzyme. The luciferase reporter plasmid for *CLDN1* was purchased from Addgene (#46387).

### Bisulfite sequencing

The genomic DNA of cell lines was extracted by DNeasy Blood & Tissue kit (Qiagen). Bisulfite conversion of genomic DNA performed by MethylCode bisulfite conversion kit (Invitrogen). The Bisulfite treated DNA was constructed into TA plasmid by specific bisulfite sequencing primers. The TA constructs were used for DNA sequencing. The bisulfite sequencing primers were designed from the MethPrimer website. The primers are listed in Table S2.

### Methylation-specific PCR

Methylation-specific PCR was performed by the Bisulfite-treated genomic DNA and methylation-specific primers. The primers were designed from the MethPrimer website. The primers are listed in Table S2.

### Pyrosequencing of CpG regions

Bisulfite-treated genomic DNA was amplified to two amplicons and was analyzed by three sequencing primers. All primers were designed using PyroMark Assay Design software and listed in Table S2. The Assay Setup and Run Setup were set by the CpG assay of PyroMark Q24 software according to the sequence of the *CLDN1* promoter. The bisulfite treatment controls were included in the program during pyrosequencing. The single-strand DNA was separated according to the manufacturer's protocols and performed the sequencing by PyroMark Q24 machine. The data were analyzed by PyroMark Q24 software and got the methylation percentage for every CpG dinucleotide.

### RNA extraction and reverse-transcription quantitative PCR (RT-qPCR)

Total cellular RNA was extracted by TRIzol solution (Invitrogen) based on the manufacturer's procedures and subjected to reverse transcription to yield cDNA using SuperScript III Reverse Transcriptase (Invitrogen), RNase Out (Invitrogen), dNTPs, and random primers. RT-qPCR was performed using the SYBR Green Master Mix (Applied Biosystems). If the cells were transfected with plasmids to overexpress protein, the extracted RNA would be treated with DNase I to remove the plasmid contamination prior to reverse transcription. The data were analyzed by the StepOne software v2.3 (three technical replicates per experiment). The primers of RT-qPCR are listed in Table S2.

### Co-immunoprecipitation

Lipofectamine 2000 (Invitrogen) was used to overexpress HA-CLDN1 and/or EPHB6-myc in HEK293T cells. Transfected cells were lysed in IP lysis buffer [100 mM NaCl, 1% (v/v) NP-40, 100 μM Na_3_VO_4_, 50 mM NaF, 30 mM sodium-pyrophosphate, and 20 mM Tris-HCl pH 7.5] and the protein concentration was measured by the method of Bradford (Bio-Rad protein assay). The total lysate of 0.5 mg was used to interact with anti-myc antibody or mouse IgG and the bound proteins were purified by PureProteome protein G magnetic beads (Merck Millipore). The nonspecific binding was washed in IP lysis buffer. The proteins specifically bound to the antibody-beads complex were eluted by 2× sample buffer. The supernatants were used to perform immunoblotting to detect HA-CLDN1 with anti-HA.

### Chromatin immunoprecipitation (ChIP)

Briefly, the proteins and DNA of cells were cross-linked by 0.75% formaldehyde and stopped the reaction by 125 mM glycine in the culture dish. Cells were scrapped and then lysed in lysis buffer [140 mM NaCl, 1 mM EDTA, 1% (v/v) Triton X-100, 0.1% (w/v) sodium deoxycholate, 0.1% (v/v) SDS, protease inhibitor, and 50 mM HEPES-KOH pH 7.5]. The DNA fragmentation was performed by EZ-zyme Chromatin Prep kit (Merck Millipore) according to the manufacturer's protocols. After DNA fragmentation, the antibodies were incubated with the supernatant at 4 °C overnight and then precipitated by Magna ChIP protein-G magnetic beads (Merck Millipore), pre-adsorbed with herring sperm DNA with bovine serum albumin. The nonspecific binding was washed in wash buffer (150 mM NaCl, 0.1% SDS, 1% Triton X-100, 2 mM EDTA pH 8.0, and 20 mM Tris-HCl pH 8.0) for three times and in final wash buffer (500 mM NaCl, 0.1% SDS, 1% Triton X-100, 2 mM EDTA pH 8.0, and 20 mM Tris-HCl pH 8.0) for once. The protein-DNA complex of specific binding was eluted by elution buffer (1% SDS and 100 mM sodium bicarbonate). The supernatant was incubated with proteinase K at 60 °C for 1 h and then reversed cross-linking at 65 °C overnight. Before DNA precipitation by QIAquick PCR purification kit (Qiagen), the samples were incubated with RNase A at 65 °C for 1 h. The purified DNA was used to perform end-point PCR or qPCR and analyzed the interesting sequence amplified by specific primers. The ChIP primers are listed in Table S2.

### shRNAs, production of lentivirus, and infection of cells

The shRNAs targeting CLDN1(TRCN0000117333 and TRCN0000117334), SLUG (TRCN0000015389), EPHB6 (TRCN0000235451 and TRCN0000235452) and RUNX3 (TRCN0000235674 and TRCN0000235675) were purchased from the National RNAi Core facility of Academia Sinica, Taiwan. The siSLUG1 (Plasmid #10903) and siSLUG2 (Plasmid #10904) were purchased from Addgene. The control shRNAs targeting LacZ (TRCN0000072224) and Luc (TRCN0000072246) were used as negative control and also purchased from the National RNAi Core facility of Academia Sinica. The shRNA sequences are shown in Table S2. To produce the lentivirus-based shRNA, seed 4 × 10^6^ of HEK293T cells in a 10 cm culture dish for 24 h and then co-transfected with 5 μg pLKO-shRNA, 5 μg pCMVΔR8.91 and 0.5 μg pMD.G in Opti-MEM by Lipofectamine 2000. Transfection medium was changed to complete medium (containing 10% FBS). Replace with 6 mL medium every day. Medium with the virus was collected at 24, 48, 72 h post-transfection and stored at 4 °C temporarily. Centrifuge supernatants at 450 × *g* for 5 min at 4 °C to remove pellet debris. The virus was filtered by a 0.45 µm low-protein-binding filter and stored in 1 mL aliquots at -80 °C to minimize freeze-thaw cycles. For infection of cells with lentivirus, the cells were seeded in a 6-well plate and incubated for 24 h before infection. The different amount of virus (multiplicity of infection = 1, 2 and 3) was mixed with the cells in 1 mL fresh medium, containing polybrene to a final concentration of 8 μg/mL. After incubated for 24 h, the medium was changed into a freshly complete medium with appropriately selective drugs to establish stable cell lines. The knockdown efficiency of genes was confirmed by RT-qPCR.

### Immunoblotting

The cells were washed twice with cold PBS and lysed in RIPA lysis buffer (150 mM NaCl, 1% (v/v) NP-40, 1% (w/v) sodium deoxycholate, 0.1% (w/v) SDS, and 25 mM Tris-HCl pH 7.6) containing a protease inhibitor cocktail (Roche) and 1 mM NaF and 1 mM Na_3_VO_4_. The supernatant was taken after centrifugation at 14,000 × *g* for 20 min. The concentration of total protein in each lysate was measured by the method of Bradford (Bio-Rad protein assay). Proteins in cell lysates were subjected to SDS-PAGE (8-12% polyacrylamide) and electrophoretically transferred to a polyvinylidene difluoride membrane (Millipore). Membranes were probed with the relevant primary antibody followed by the appropriate horseradish peroxidase-conjugated secondary antibody (Jackson ImmunoResearch). Immunopositive signals were detected by chemiluminescence (PerkinElmer).

### Migration assay

The cells were seeded in top well of trans-well (8 μm pore, Costar 3422) with medium contained 1% serum and the medium with 10% serum was added into the bottom well. After incubation of cells for 16 h, the non-migrated cells were erased with a cotton swab, and migrated cells were stained with ASK Liu's stain A and B solution. Each entire trans-well was imaged with five fields. The migrated cells were counted by software, ImageJ.

### Sphere assay

The cells were seeded in a 6-well plate coated with poly-HEMA (Sigma) or in the ultra-low-attach plate (Corning). The cells were incubated in serum-free medium with 1× N2 supplement, 20 ng/mL bovine fibroblast growth factor and 20 ng/mL epidermal growth factor for 10 days. The number of spheres was counted under the microscope. The diameter of the sphere > 100 μm was counted.

### Immunofluorescence by confocal microscopy

The transfected cells were seeded onto the glass coverslips for 24 h. The cells were washed with PBS twice, fixed with 4% formaldehyde for 10 min at room temperature, and permeabilized with 0.1% (v/v) Triton X-100 for 10 min at room temperature. The cells were blocked with 5% bovine serum albumin in PBS for 30 min. After washed with PBS, the cells incubated with the monoclonal anti-myc antibody at 4 °C overnight followed by the Dylight 594-conjugated secondary antibody (Abcam) for 1 h. The cells were mounted and counterstained with 4',6-diamidino-2-phenylindole (DAPI) by ProLong Gold antifade reagent (Invitrogen). Images were obtained with a confocal laser-scanning microscope (NIKON EZ-C1).

### Immunohistochemistry

The tumor samples were paraffin-embedded and cut into 5 μm-thick sections. The tissues were de-paraffinized in xylene and hydrated through a graded series of alcohol. Antigen retrieval was performed using 10 mM sodium citrate (pH 6.0), microwaved for 15 min. The tissues were then blocked in 3% hydrogen peroxide for 15 min at room temperature. The target proteins were detected with antibodies, including CLDN1 (1:25; Invitrogen), EPHB6 (1:500, Sigma), SLUG (1:150, Santa Cruz). The primary antibodies were incubated for overnight at 4 °C. The detection was performed using the Starr Trek Universal HRP Detection kit (Biocare Medical). Tissues were counterstained with hematoxylin and mounting.

### Luciferase reporter assay

The cells were seeded in 24-well plates and co-transfected with plasmids for 16 h, containing 250 ng of pGL4.1-*CLDN1* promoter constructs (expressing luciferase), 250 ng of the RUNX3 expression plasmid or vector, and 5 ng pRL-*Renilla* (Promega) as a normalization control. The luciferase activity was determined with the dual luciferase assay system (Promega) by a luminometer (Thermo, luminoskan ascent). Each condition was performed in three biological experiments.

### Cytotoxicity

The cells were seeded in 96 well-plate to the cell number of 3,000 per well for 24 h prior to drug treatment. The cells were treated with the anticancer drug at a different concentration for 72 h. The cell viability was measured by WST-1. The IC_50_ was calculated by CalcuSyn software. For the two-drug combination experiments, the CL_1-5_ cells were treated with the serial concentrations of cisplatin which combined with three concentrations of TSA or SAHA, including IC_25_, IC_50_, IC_75_ of TSA or SAHA for CL_1-5_. Simultaneously, the cells were treated with the serial concentrations of TSA or SAHA which combined with three concentrations of cisplatin, including IC_25_, IC_50_, IC_75_ of cisplatin for CL_1-5_. The cell viability was measured by WST-1 and the combination index was calculated by CalcuSyn software.

### Flow cytometry for detection of CD133, determination of cell-cycle stages, and apoptosis

The CD133/2 (293C3)-PE antibodies (Miltenyi Biotec) were used to detect the CD133 membrane expression by flow cytometry (BD FACSCanto II, BD biosciences) according to the manufacturer's protocols. The FITC-Annexin V/PI apoptosis detection kit (BD) was used to detect the percentage of the apoptotic cell by flow cytometry (BD FACSCanto II) according to the manufacturer's protocols. In addition, propidium iodide (PI) staining was used to analyze the cell cycle by flow cytometry (BD FACSCanto II).

### Aldefluor assay

Cellular ALDH activity was measured by the Aldefluor assay (Stemcell Technologies) according to the manufacturer's protocols. Briefly, the cells were detached from the culture dish, washed twice with PBS and resuspended in a cell concentration of 1 × 10^6^ per mL. Activated Aldefluor reagents (5 μL) were added into the 1 mL cell sample in a test tube, mixed and immediately transferred into 0.5 mL of cell suspensions to the control tube with 5 μL DEAB reagent. Post two tubes were incubated for 30 min at 37 °C, cell suspensions were centrifugated for 5 min at 250 × *g* and then supernatants were removed. Finally, cell pellets were resuspended in 0.5 mL of the Aldefluor assay buffer and stored on ice until the flow assay was performed. The percentage of ALDH^+^ cells were measured by Fluorescence channel (FITC) vs. SSC in dot plots using flow cytometry (BD FACSCanto II). The background of fluorescence was set using the cells of the control tube.

### Soft-agar colony formation

The sterile 0.5% base agar in the RPMI medium containing 10% FBS was prepared and solidified in a 6-well plate. The sterile 0.35% top agar in RPMI medium containing 10% FBS was prepared and mixed with a cell number of 500 per well and then immediately plated on the top of base agar. After the top agar is solidified, the soft agar plate was incubated at 37 °C until the colony is visible under the microscope. The fresh medium was added to the wells twice every week. For counting the colony, the cell colonies were fixed with 3.7% paraformaldehyde for 30 min at room temperature and stained with 0.01% crystal violet. The diameter of the colony over 100 μm was counted under the dissecting microscope.

### Animal experiments

For tumor initiation assay, the mice were randomly divided into two groups. The cancer cells harboring the vector or CLDN1 overexpression were subcutaneously implanted into 6-week-old non-obese diabetic-severe combined immunodeficiency (NOD-SCID) mice (*n* = 6 per group). For cisplatin sensitivity experiments, the mice were randomly divided into two groups. The cancer cells harboring the CLDN1 overexpression or vector were subcutaneously implanted into the same 6-week-old NOD-SCID mice on days 1 and 8, respectively, and the mice were administrated with 2.5 mg/kg cisplatin, twice every week from day 15 (*n* = 8 per group). For the frequency of CSCs estimated by extreme limiting dilution analysis, the mice were randomly divided into four groups, Hop62/shLuc-shLuc, Hop62/shC34-shLuc, Ho62/shLuc-shSLUG-S3, and Hop62/shC34-shSLUG-S3, including vector, the knockdown of CLDN1 and/or SLUG. The NOD-SCID mice had subcutaneously implanted four different cell densities, 5 × 10^5^, 5 × 10^4^, 5 × 10^3^ and 5 × 10^2^, for every group (*n* = 6 or 8 per group for one cell density). The frequency of CSCs was calculated by the extreme limiting dilution analysis website. The humane endpoint of the experiments was that the tumor volume reached ~1,500 mm^3^. The mice were sacrificed, and tumor mass or lung organs were removed and fixed in 10% formalin. The tumor nodules on the surface of the lung were counted. The lung tissue was embedded in paraffin, sliced into 5 μm for hematoxylin and eosin staining. The volume of the tumor was measured by Vernier caliper and the tumor weight was measured by an electronic balance.

### Data availability

The CLDN1-overexpressing cDNA microarray data (GSE10309) were downloaded from Gene Expression Omnibus (GEO) and used to analyze the singling pathway. Gene expression data from the TCGA Lung Adenocarcinoma (TCGA-LUAD) database were downloaded by the UCSC Xena browser and used to examine *CLDN1* expression in different stages, the correlation between *CLDN1* expression and stemness score, and the correlation between *CLDN1* and *EPHB6* expression. The data of *CLDN1* or *EPHB6* expression, level 3 RNA-seq data, were represented as log2*(x +* 1) transformed RSEM (RNA-Seq by Expectation Maximization) normalized count. The GSE27262 were downloaded from GEO and used to analyze the *CLDN1* expression between normal and tumor paired samples. The Kaplan-Meier Plotter website and previously published microarray datasets, GSE31210 and GSE68465, were used to analyze the Kaplan-Meier estimation among the different patient groups based on the expression of *CLDN1*, *RUNX3*, and *SLUG*. All other data that support the findings of this study are available from the corresponding author on reasonable request.

### Statistics and reproducibility

Data are presented as the mean ± s.d. or mean ± s.e.m. The unpaired two-sided Student *t*-test was used to compare the difference between two groups for continuous variables. The one-way ANOVA was used to compare the difference among multiple groups, following post-hoc comparisons by Duncan's method. The Spearman correlation test was used to analyze the correlation between the two gene expression because of the small sample size. The pairwise tests for differences in stem-cell frequencies were determined by the chi-squared test. Kaplan-Meier plots were used to assess survival, and the log-rank test was used to compare the differences between survival curves. All pairwise multiple comparisons were performed by the Holm-Sidak method. Statistical analyses were performed with SigmaPlot and GraphPad Prism. The *p* values for statistical significance are presented as: *p < 0.05, **p < 0.01, ***p < 0.001. All *n* values and reproducibility were indicated in the figure legends.

### Study approval

All animal experiments were conducted according to the guidelines of the Laboratory Animal Center and were approved by the IACUC of the National Cheng Kung University, Taiwan.

## Results

### CLDN1 inhibits cell migration by repressing SLUG expression through ERK1/2

**Figure 1:**
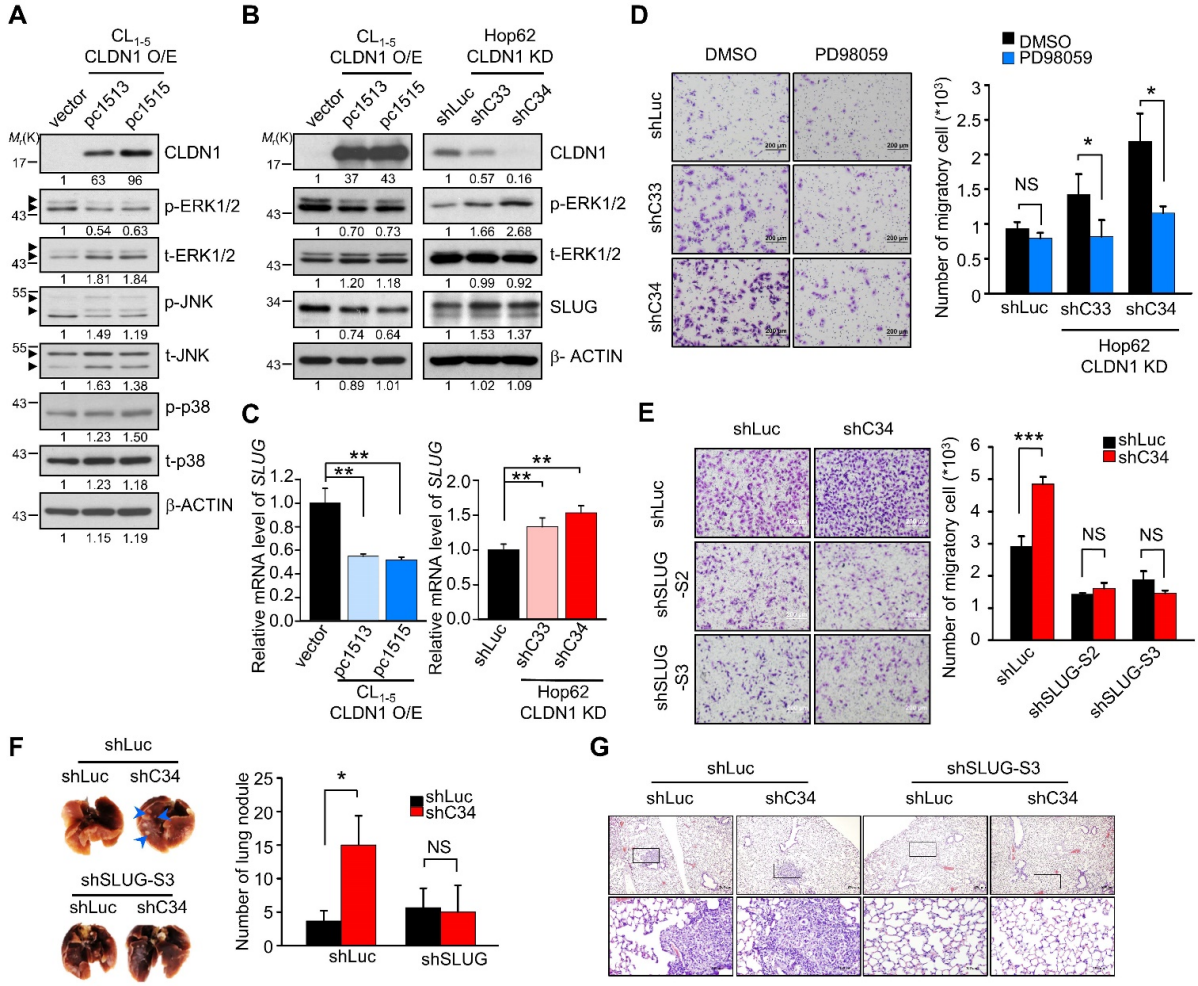
** CLDN1 inhibits cell migration by repressing SLUG expression through ERK1/2. (A)** The phosphorylated levels of MAPKs and CLDN1 in CL_1-5_ with vector or CLDN1 overexpression (CL_1-5_ CLDN1-O/E) were measured by immunoblotting. **(B)** The levels of CLDN1, phospho-ERK1/2 and SLUG in CL_1-5_ cells overexpressing CLDN1 or in Hop62 cells in which CLDN1 was knocked down (Hop62 CLDN1-KD) were measured by immunoblotting. **(C)** The mRNA level of *SLUG* was analyzed by RT-qPCR in CLDN1 O/E or KD cells (three technical replicates per experiment). **(D)** Hop62 shLuc and CLDN1-KD cells were pretreated with DMSO or 5 μM PD98059 for 6 h. Then, the cell migration was measured by transwell chambers for 16 h under the medium condition with DMSO or 5 μM PD98059. Shown are representative images (left) and quantification of cell migration (right) (*n* = three biologically independent experiments). **(E)** The cell migration was measured in Hop62 cells with knockdown of CLDN1 and/or SLUG (*n* = three biologically independent experiments). Shown are representative images (left) and quantification of cell migration (right).** (F and G)** The metastasis potential was estimated by xenograft. Hop62 stable cell lines, shLuc-shLuc, shC34-shLuc, shLuc-shSLUG-S3, and shC34-shSLUG-S3 subcutaneously inoculated in NOD-SCID mice (*n* = 3 mice per group). Shown are representative images** (F, left)**, quantification of metastatic lung nodule** (F, right)**, and hematoxylin and eosin (H&E) staining** (G)**. The arrows indicated the metastatic lung nodules. Error bars indicated in **C, D, E** and** F** represent the mean ± s.d. *p < 0.05, **p < 0.01 and ***p < 0.001 (two-tailed Student's* t*-test). NS: non-significant. β-ACTIN serves as the loading control in immunoblots.

Overexpression of CLDN1 suppresses the metastasis of lung adenocarcinoma 18, but the underlying mechanism remains unclear. To investigate how CLDN1 influences cell migration and metastasis, we used our CLDN1-overexpressing cDNA microarray data (GSE10309) to analyze the singling pathway using MetaCore software, revealing that CLDN1 could modulate MAPK signaling, EMT and cell adhesion (Figure S1A). The basal levels of CLDN1 in lung cancer cell lines were detected by RT-qPCR (Figure S1B) and showed that Hop62 is high CLDN1-expressing cells, and CL_1-5_ is low CLDN1-expressing cells. Therefore, we used Hop62 cells to knock down *CLDN1* and CL_1-5_ cells to overexpress *CLDN1*. The phosphorylation of MAPK family members (ERK1/2, JNK, and p38) was measured in CLDN1-overexpressing CL_1-5_ cells. The phosphorylation of ERK1/2 was downregulated in the cells overexpressing CLDN1 (Figure 1A), and CLDN1 overexpression also suppressed ERK1/2 activation upon stimulation with serum (Figure S1C). Moreover, we silenced CLDN1 in Hop62 cells by lentiviruses that produced two different short hairpin RNAs (shRNAs) targeting CLDN1 (shC33 and shC34) and found that CLDN1 knockdown increased the phosphorylation of ERK1/2 (Figure S1D). Phosphorylation of JNK was slightly decreased in CLDN1-overexpressing cells but not changed in CLDN1 knockdown cells (Figure S1E). Besides, the phosphorylation of p38 showed no change in CLDN1-overexpressing cells (Figure 1A). These results revealed that CLDN1 downregulates ERK1/2 signaling.

The transcription factors (TFs) SLUG, SNAIL, TWIST, and ZEB contribute to EMT and cancer progression 37. Dysregulated expression of SLUG promotes metastasis in lung cancer 38, 39 and can be regulated by ERK1/2 signaling 40. The expression of these EMT-TFs in CLDN1-silenced or overexpressed cells were checked by RT-qPCR or Western blot (Figure S1F and Figure S1G). The results showed that the levels of SLUG were inversely correlated with the levels of CLDN1 but others EMT-TFs not. We treated CL_1-5_ cells with the MEK1/2 inhibitor PD98059 to confirm that downregulation of ERK1/2 signaling could suppress SLUG expression (Figure S1H). Then we showed that CLDN1 overexpression in CL_1-5_ cells decreased the levels of both SLUG mRNA and protein, accompanied by the downregulation of phosphorylated ERK1/2. Conversely, CLDN1 knockdown in Hop62 cells increased the levels of SLUG mRNA and protein, accompanied by upregulation of phosphorylated ERK1/2 (Figure 1B-C). Therefore, SLUG may be the metastasis-related effector regulated by CLDN1 through ERK1/2 signaling.

Hop62 control (shLuc) and CLDN1 knockdown (shC33 and shC34) cells were treated with PD98059, with subsequent assessment of cell migration. The results showed that PD98059 attenuated the CLDN1 knockdown-induced increase in cell migration. (Figure 1D). Therefore, the loss of CLDN1 increased cell migration through ERK1/2 signaling. Furthermore, we knocked down SLUG using lentiviruses encoding different shRNAs in Hop62 shLuc and shC34 cells (Figure S1I) and found that SLUG silencing abrogated the CLDN1 knockdown-mediated increase in cell migration (Figure 1E). Importantly, silencing of SLUG also abrogated the lung metastasis promoted by CLDN1 loss *in vivo* (Figure 1F-G). For long-term observation of cancer metastasis, knockdown of SLUG abrogated the lung metastasis promoted by CLDN1 loss (Figure S1J). Taken together, these results demonstrated that CLDN1 inhibits SLUG expression via suppression of the ERK1/2 pathway to inhibit cancer-cell migration and metastasis.

### CLDN1 upregulates EPHB6 to inhibit ERK1/2 signaling and cell migration

**Figure 2:**
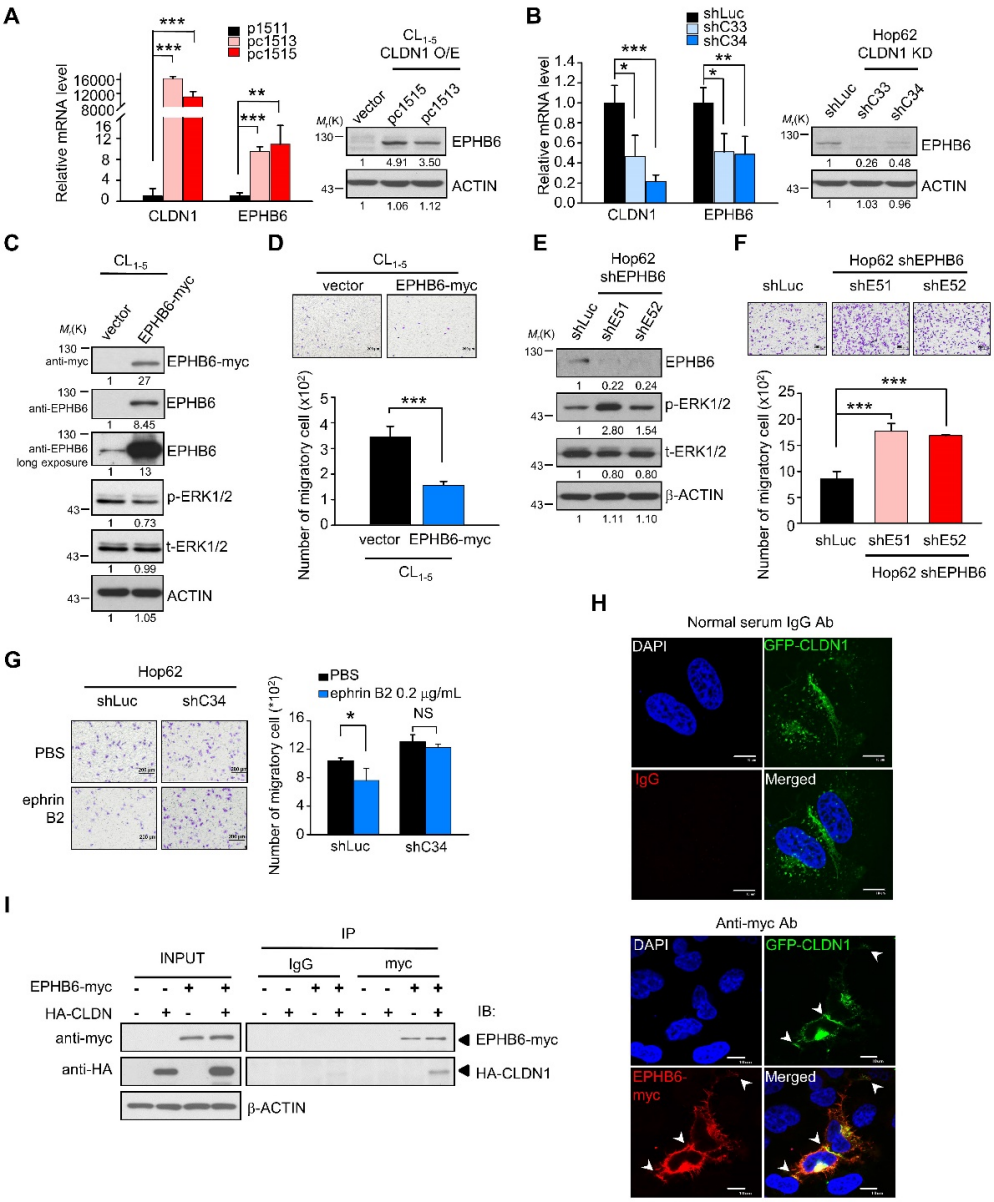
** CLDN1 upregulates EPHB6 to inhibit ERK1/2 signaling and cell migration. (A and B)** The mRNA and protein levels of EPHB6 were respectively measured by RT-qPCR and immunoblotting in cells overexpressing CLDN1** (A, pc1513 and pc1515)** or with CLDN1 knockdown** (B, shC33 and shC34)** (three technical replicates per experiment). **(C)**The phosphorylation of ERK1/2 and other indicated proteins was measured by immunoblotting in CL_1‑5_ cells overexpressing EPHB6 (EPHB6-myc). **(D)** The cell migration of CL_1‑5_ overexpressing EPHB6 was measured by transwell chambers. Shown are representative images (top) and quantification of cell migration (bottom). **(E)** Phosphorylation of ERK1/2 was detected by immunoblotting in Hop62 cells with EPHB6 knockdown (shE51 and shE52). **(F)** The cell migration of EPHB6-knockdown Hop62 was measured. Shown are representative images (top) and quantification of cell migration (bottom). **(G)** The migration was performed when Hop62 cells with knockdown of CLDN1 (shC34) or shLuc were treated with 0.2 μg/mL ephrin B2 or PBS. Shown are representative images (left) and quantification of cell migration (right). **(H)** The immunofluorescence (IF) showed the localization of CLDN1 and EPHB6 by confocal microscopy. The arrows indicated the colocalization of CLDN1 and EPHB6 in tight junction.** (I)** The interactions of CLDN1 and EPHB6 were examined by the co-immunoprecipitation (IP) assay using the anti-myc antibody in cells ectopically overexpressed EPHB6 and CLDN1. The *n* values in **D, F,** and** G** were three biologically independent experiments. Error bars indicated in **A, B, D, F,** and** G** represent the mean ± s.d. *p < 0.05, **p < 0.01, ***p < 0.001 (two-tailed Student's *t*-test). NS: non-significant. β-ACTIN serves as the loading control in immunoblots. The input served as the loading control in IP assay. IgG serves as specific binding control in IP or IF assay.

Because CLDN1 is a membrane protein, we speculated that CLDN1 might cooperate with some membrane protein (such as receptor tyrosine kinases, RTK) to inhibit the ERK1/2 pathway. In samples from patients with non-small-cell lung cancer, Muller-Tidow *et al*. determined the risk of distant metastasis based on receptor tyrosine kinase expression and identified EPHB6 as a strong suppressor of metastasis 41. Accordingly, we analyzed our cDNA microarray data (GSE10309) 22 with respect to the expression of receptor tyrosine kinases, which inversely correlated with distant metastasis shown by Muller-Tidow *et al*. The eight RTKs of distant-metastasis suppressors were analyzed in CLDN1-overexpressing cells. Only the EPHB6 gene is up-regulated in CLDN1-overexpressed cells (Figure S2A), while the expression of other RTKs is no change. CLDN1 overexpression upregulated the levels of both EPHB6 mRNA and protein (Figure 2A), whereas CLDN1 knockdown downregulated EPHB6 levels (Figure 2B). Moreover, overexpression of EPHB6 in CL_1-5_ cells suppressed the phosphorylation of ERK1/2 and cell migration (Figure 2C-D), whereas knockdown of EPHB6 in Hop62 cells promoted ERK1/2 signaling and cell migration (Figure 2E-F). We also found that the EPHB6 ligand, ephrin B2 (EFNB2), could inhibit phosphorylation of ERK1/2 and the migration of CLDN1-expressing Hop62 cells but not that of CLDN1-silenced cells (Figure 2G and Figure S2B). Hence, EPHB6 signaling was necessary for CLDN1-mediated suppression of ERK1/2 activity and cell migration.

Finally, we used confocal microscopy to assess the subcellular localization of CLDN1 and EPHB6 in CL_1-0_ cells co-expressing GFP-CLDN1 and EPHB6-myc. CLDN1 and EPHB6 co-localized predominantly at cell junctions (Figure 2H), and co-immunoprecipitation confirmed this interaction (Figure 2I). These results demonstrated that CLDN1 upregulated and then interacted with EPHB6 at cell junctions, thereby inhibiting ERK1/2 signaling and cell migration.

### CLDN1 represses the CSC phenotype and sensitizes lung adenocarcinoma cells to chemotherapy drugs *in vitro.*

CLDN1-deficient breast cancer cell subpopulation reflects a CSC phenotype 42. SLUG also has been demonstrated to promote the generation of CSCs 39 and resistance to chemotherapy 43. Here, we first analyzed the correlation between the stemness score 44 and CLDN1 expression in the TCGA database. The data showed CLDN1 expression negatively correlated with stemness score in TCGA-LUAD cohort (Figure S3A) (*r* = -0.256, *p* = 0.0000000043) implying that CLDN1 expression might repress stemness property. Hence, we next explored whether the loss of CLDN1 induces the formation of CSCs or drug resistance via SLUG in lung adenocarcinoma.

**Figure 3:**
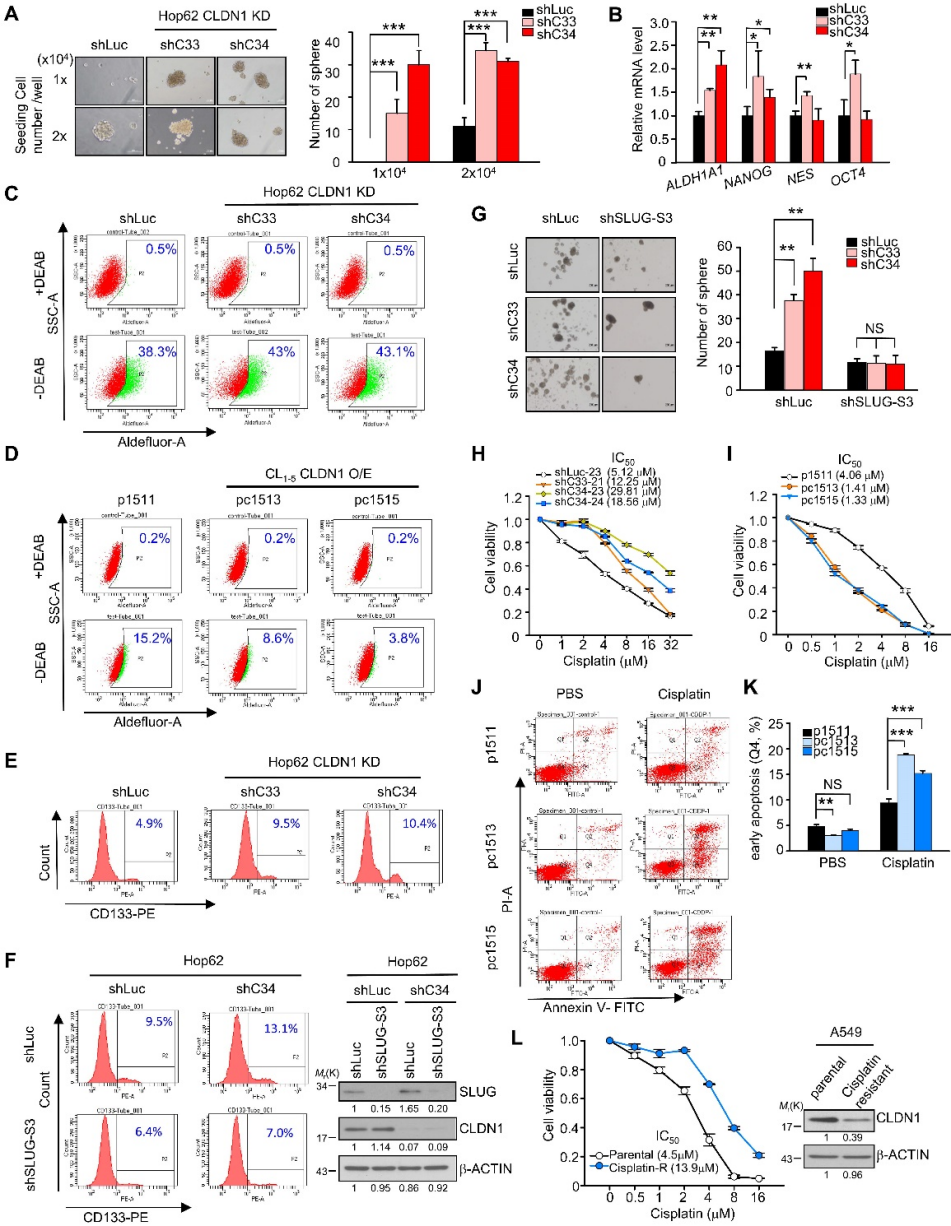
**CLDN1 represses the CSC phenotype and sensitizes lung adenocarcinoma cells to cisplatin *in vitro*. (A)** Hop62 with CLDN1 silence (shC33 and shC34) were performed in sphere assay to evaluate the population of CSCs. The spheres (the diameter is over 100 μm) were counted. Representative images (left) and quantified data (right) are shown. **(B)** The markers of stemness, including *ALDH1A1*, *NANOG*,* NES*, and *OCT4* were measured by RT-qPCR in Hop62 cells with CLDN1 knockdown (shC33 and shC34) (three technical replicates per experiment). **(C and D)** ALDH activity was measured by Aldefluor assay in the cells with CLDN1 knockdown** (C, shC33 and shC34)** or overexpression **(D, pc1513 and pc1515)**. The diethylaminobenzaldehyde (DEAB) is an inhibitor of ALDH. **(E)** The percentage of CD133^+^ cells was estimated by flow cytometry in Hop62 cells with CLDN1 knockdown (shC33 and shC34).** (F)** The percentage of CD133^+^ Hop62 cells with knockdown of CLDN1 (shC34) and/or SLUG (shSLUG-S3) was estimated by flow cytometry (left). Immunoblotting indicated a successful knockdown of CLDN1 and SLUG (right). **(G)** The sphere assay was used to estimate the property of CSCs in Hop62 cells with CLDN1 knockdown (shC33 and shC34) and/or SLUG knockdown (shSLUG-S3).** (H and I)** The cytotoxicity of cisplatin was measured in the cells with CLDN1 knockdown** (H, shC33 and shC34)** or overexpression** (I, pc1513 and pc1515)**. IC_50_: half maximal inhibitory concentration.** (J and K)** The percentage of cell death and early apoptosis in cisplatin-treated CL_1-5_ cells overexpressing CLDN1 (pc1513 and pc1515) was evaluated by the annexin V/PI assay. Shown are representative images (**J**) and quantification of the percentage of early apoptosis **(K)**. **(L)** A549 cells were trained as cisplatin-resistant cell line (left) and then the expression of CLDN1 was measured by immunoblotting (right). The *n* values in **A, G, H, I, K** and** L** were three biologically independent experiments. Error bars indicated in** A, B, G** and** K** represent the mean ± s.d. Error bars indicated in** H, I** and** L** represent the mean ± s.e.m. *p < 0.05, **p < 0.01, ***p < 0.001 in** A, B, G** and** K** (two-tailed Student's *t*-test). NS: non-significant. β-ACTIN serves as the loading control in immunoblots.

We found that CLDN1 knockdown increased sphere formation (only the CSCs can form the sphere; Figure 3A), SLUG expression (in sphere culture condition; Figure S3B), and stemness markers including *ALDH1A1*, *NANOG*, *NES*, and *OCT4*; notably, *ALDH1A1*, aldehyde dehydrogenase (ALDH) 1 family member A1, (also prognostic and stemness marker for lung adenocarcinoma) 45, 46 was upregulated to a greater extent than the other markers (Figure 3B). The percentage of ALDH^+^ cells was increased upon CLDN1 knockdown (Figure 3C: representative figure; Figure S3C: quantified data), whereas ectopic expression of CLDN1 decreased the population of ALDH^+^ cells (Figure 3D: representative figure; Figure S3D: quantified data). Consistent with these results, CLDN1 knockdown also increased the population of CSCs expressing CD133^+^, another marker of CSCs in lung cancer 45, 47 (Figure 3E). The loss of CLDN1 could enrich the population of ALDH^+^ or CD133^+^ CSCs. Next, to investigate whether SLUG mediates the enrichment of CSCs caused by loss of CLDN1, the CD133^+^ population and sphere formation were measured in Hop62 cells in which CLDN1 and/or SLUG was knocked down. SLUG knockdown dramatically blocked the CLDN1 knockdown-enhanced CD133^+^ population and sphere formation (Figure 3F-G).

Compared with primary-tumor cells, CSCs are more resistant to chemotherapy drugs 7, 48, and SLUG has an antiapoptotic effect through suppression of PUMA 49, 50. In this respect, CLDN1-silenced Hop62 cells became more resistant to cisplatin, carboplatin, and taxol (Figure 3H, Figures S3E and S3F). Conversely, CLDN1 overexpression sensitized cancer cells to cisplatin, carboplatin, and taxol (Figure 3I and Figure S3G). Among cisplatin-treated CL_1-5_ cells, those overexpressing CLDN1 were killed more easily (Q2+Q4) or were more prone to early apoptosis (Q4) (Figure 3J-K and Figure S3H) further, the proportion of G2/M-phase cells increased (Figure S3I), which enhances cisplatin response 51. Moreover, to evaluate whether cisplatin-resistance cells showed low CLDN1 expression, we got the cisplatin-resistant A549 cells by treating cancer cells with slowly increased the concentration of cisplatin for six months. The results showed CLDN1 expression was downregulated in cisplatin-resistant A549 cells compared with parental cells (Figure 3L). These findings demonstrated that the enrichment of CSCs caused by loss of CLDN1 was mediated through SLUG upregulation. CLDN1 overexpression may thus sensitize cancer cells to cisplatin by inhibiting the percentage of CSCs and enriching the percentage of cells in the G2/M phase.

### CLDN1 represses tumorigenesis and CSC properties and sensitizes lung adenocarcinoma to chemotherapy drugs *in vivo.*

**Figure 4:**
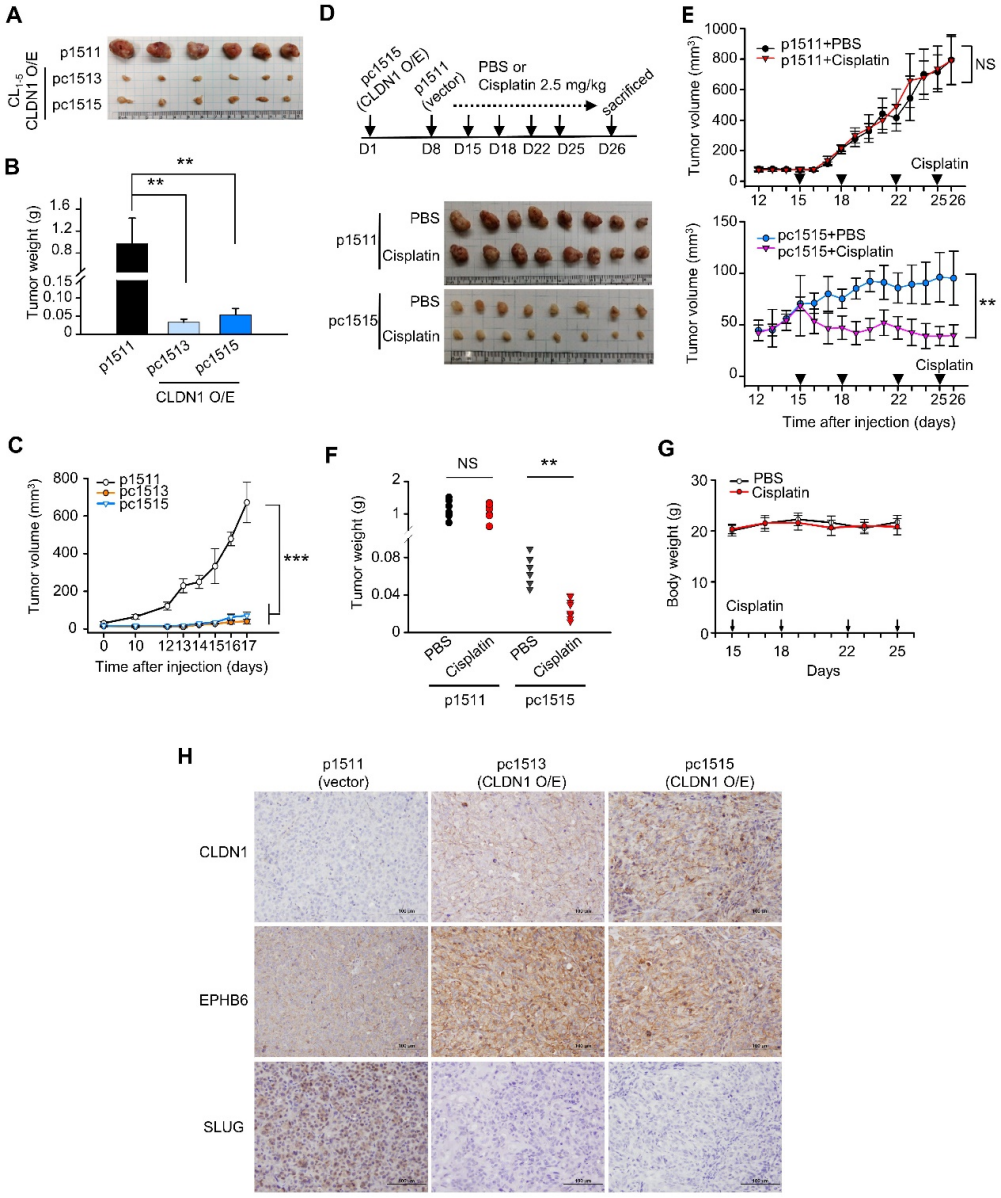
** CLDN1 represses tumor growth and CSC properties and sensitizes lung adenocarcinoma to cisplatin *in vivo.* (A-C)** The tumor xenograft showed the tumorigenesis of CL_1-5_ cells with vector (p1511) or CLDN1 overexpression (pc1513 and pc1515). *n* = 6 mice per group. The mice were sacrificed at Day 17 and got the tumor mass. The analysis of tumor mass** (A)**, tumor weight **(B)**, and tumor volume** (C)** are shown. **(D-G)** On day 1 of the experiment, pc1515 cells were injected subcutaneously into the right posterior flank each NOD-SCID mice; on day 8, p1511 cells (as a control) were injected subcutaneously into the left posterior flank. Tumor volume was similar in the two flanks on day 15. On day 15, the mice then received cisplatin (intraperitoneal, 2.5 mg/kg) or phosphate-buffered saline (PBS, control) on days 15, 18, 22 and 25 and were sacrificed at day 26 and got the tumor mass. The schedule of cisplatin treatment and tumor mass **(D)**, tumor volume** (E)**, tumor weight **(F)**, and body weight **(G)** of the tumor-bearing mice are shown (n = 8 mice per group). **(H)** The IHC experiment was used to evaluate the CLDN1, EPHB6, and SLUG expression* in vivo*. Error bars indicated in** B, C, E** and** G** represent the mean ± s.d. **p < 0.01, ***p < 0.001 in** B, C, E** and** F** (two-tailed Student's *t*-test). NS: non-significant.

As shown in Figure S4A-B, CLDN1 overexpression decreased CL_1‑5_ cell proliferation and colony formation in soft agar *in vitro*. To study the effect of CLDN1 on tumorigenesis *in vivo*, we carried out a subcutaneous xenograft assay with non-obese, severe diabetic, combined immunodeficiency (NOD-SCID) mice. CL_1-5_ cells with CLDN1 expression (pc1513 and pc1515) or vector (p1511) were subcutaneously injected into the posterior flank of male NOD-SCID mice. CLDN1 overexpression dramatically decreased tumor growth (Figure 4A-C). Next, we studied the effect of CLDN1 on the drug sensitivity of lung tumors *in vivo*. The p1511 and pc1515 cells were subcutaneously injected into a different side of the posterior flank of male NOD-SCID mice. The mice then received cisplatin (intraperitoneal, 2.5 mg/kg) or phosphate-buffered saline (PBS, control) (Figure 4D). Tumors derived from pc1515 responded to cisplatin *in vivo*, whereas tumors derived from p1511 did not (Figure 4, E-F); body weight was not affected (Figure 4G). To assess the long-term growth of pc1515-derived tumors in NOD-SCID mice treated with cisplatin, pc1515 cells were injected subcutaneously into the posterior flank of male mice, and then the mice were treated with PBS or cisplatin over 50 days. Both tumor volume and weight were significantly smaller in the cisplatin-treated mice compared with controls, and body weight was not affected (Figure S4C-F). Consistent with the *in vitro* result, these results indicated that CLDN1 sensitizes lung-cancer cells to cisplatin *in vivo*. Furthermore, we performed the immunohistochemistry (IHC) experiments by using our xenograft tumor samples to confirm the signaling *in vivo*. The results showed that CLDN1 overexpression positively correlated with EPHB6 expression and negatively correlated with SLUG expression, indicating that the CLDN1-EPHB6-SLUG axis also exists *in vivo* (Figure 4H).

Next, we investigated the effect of CLDN1 on the production of CSCs* in vivo*, and extreme limiting dilution analysis was used to evaluate the frequency of CSCs. CLDN1 knockdown (shL-C34) in Hop62 cells increased the frequency of CSCs from 1 in 2,239 (control cells, shL-L) to 1 in 361 (Table 1). In SLUG-silenced Hop62 cells (shS3-L), the frequency was 1 in 22,951, and CLDN1 knockdown (shS3-C34) did not substantially increase the frequency (1 in 24,080). These results demonstrated that loss of CLDN1 could enrich the population of CSCs and that SLUG contributes to the formation of CSCs induced by loss of CLDN1.

### DNA hypermethylation of the* CLDN1* promoter maintains its transcription by abrogating SLUG-mediated suppression

**Figure 5:**
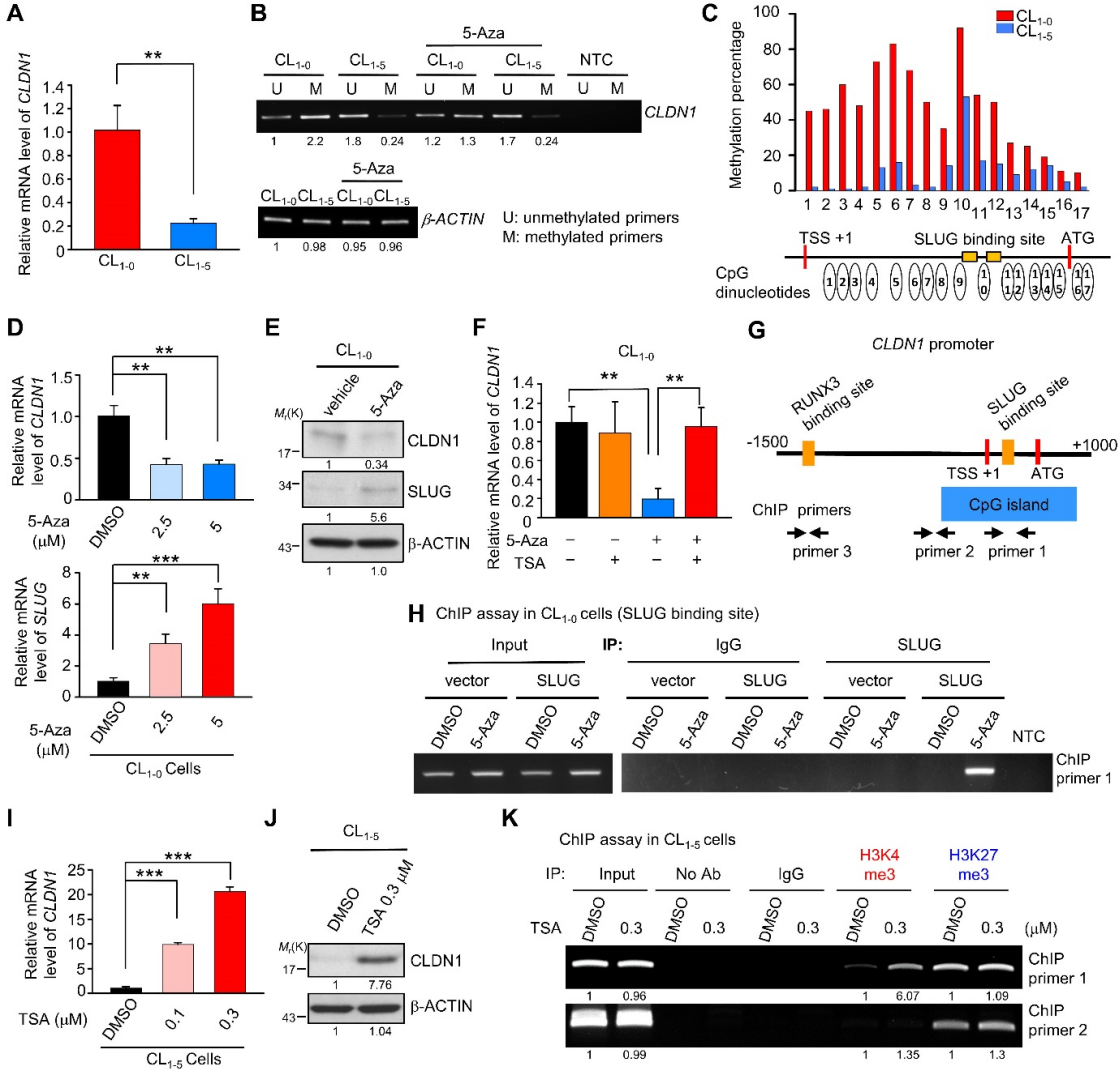
** DNA hypermethylation of the *CLDN1* promoter maintains its transcription by abrogating SLUG-mediated suppression. (A)** The mRNA levels of *CLDN1* in CL_1-0_ and CL_1-5_ cells. **(B)** The DNA samples of CL_1-0_ and CL_1-5_ cells were bisulfite-converted genomic DNA and performed PCR by specific primers. The methylation-specific PCR was used to measure the methylation patterns of the *CLDN1* promoter in CL_1-0_ and CL_1-5_ cells. The *β-ACTIN* could also serve as a positive control for MS-PCR experiments and internal control to measure DNA input. The *β-ACTIN* primer was designed in a non-CpG containing region. NTC: non-template control. 5-Aza: DNA methylation inhibitor. **(C)** The percentage of the methylation status of the *CLDN1* promoter in CL_1-0_ and CL_1‑5_ cells was quantified by the pyrosequencing assay.** (D and E)** CL_1-0_ cells were treated with 5-Aza and then the mRNA** (D)** or protein** (E)** levels of indicated genes were measured. **(F)** CL_1-0_ cells were treated with 5-Aza and/or TSA, and then the mRNA levels of *CLDN1* were measured by RT-qPCR (three technical replicates per experiment). Error bars indicate the mean ± s.d. **p < 0.01 (two-tailed Student's* t*-test). **(G)** The scheme showed the region which amplified by ChIP primer 1, 2 and 3 and the region of CpG island in the *CLDN1* promoter. TSS: transcription start site. **(H)** CL_1‑0_ cells were treated with 5-Aza and ectopically overexpressed SLUG. Subsequently, the ChIP assay was performed by anti-SLUG antibody, and the ChIP primer 1 was used to amplify the SLUG-binding site of the *CLDN1* promoter.** (I and J)** CL_1-5_ cells were treated with TSA and the mRNA **(I)** or protein** (J)** levels of CLDN1 were measured. **(K)** CL_1-5_ cells were treated with TSA and then the ChIP assay was performed using H3K4me3 or H3K27me3 antibodies and ChIP primer 1 or 2, which respectively amplified the region of SLUG-binding site or near CpG island of the *CLDN1* promoter. NTC: non-template control. The input serves as the loading control and IgG as specific binding control in the ChIP assay.

During tumorigenesis, tumor-suppressor genes are often silenced by DNA methylation and histone modifications. To investigate the mechanisms by which *CLDN1* transcription is regulated, we utilized CL_1-0_ and CL_1-5_, the well-characterized cell lines that differ in cell migration 52, in which CLDN1-EPHB6-ERK1/2-SLUG axis was active or not (Figure S5A). Also, these paired lung cancer cell lines with a similar genetic background that would be suitable to study gene regulation. The CL_1-0_ cells expressed more *CLDN1* mRNA than CL_1‑5_ cells (Figure 5A). The MethPrimer website predicted that the *CLDN1* promoter contains a CpG island containing 68 CpG sites (Figure S5B, bottom), indicating that *CLDN1* expression may be epigenetically regulated. Unexpectedly, bisulfite sequencing revealed hypermethylation of the *CLDN1* promoter in CL_1-0_ cells compared with CL_1-5_ cells (Figure S5B, top), and this was confirmed by methylation-specific PCR (Figure 5B). Further, pyrosequencing quantified 17 differentially methylated CpG sites between CL_1-0_ and CL_1-5_ cells. In CL_1-0_ cells, most of the 17 CpG sites are hypermethylation. (Figure 5C). Interestingly, these hypermethylated CpG sites contain the binding sites for SLUG, a negative regulator of *CLDN1* 53, 54. Moreover, DNA hypermethylation tends to reflect condensed DNA, which inhibits transcription-factor binding 55. An inhibitor of DNA methylation, 5-Azacytidine (5-Aza), was used to block DNA methylation in the *CLDN1* promoter in CL_1-0_ cells. 5-Aza decreased the amount of both *CLDN1* mRNA and protein in a dose-depended manner, and it increased *SLUG* expression (Figure 5D-E)*.* Additionally, trichostatin A (TSA), which inhibits histone deacetylases (HDACs), could inhibit the ability of SLUG to repress *CLDN1* transcription. Treatment of CL_1-0_ cells with 5-Aza decreased *CLDN1* expression, whereas TSA had no effect. Importantly, *CLDN1* transcription was not affected when CL_1-0_ cells were treated with both 5-Aza and TSA (Figure 5F). To investigate whether SLUG could be involved in 5-Aza-induced *CLDN1* repression in CL_1-0_ cells, chromatin immunoprecipitation (ChIP) was performed to detect SLUG binding to the *CLDN1* promoter. The regions within the *CLDN1* promoter that were detected by ChIP primers are shown in Figure 5G. After 72 h of treatment with 5-Aza, CL_1-0_ cells were ectopically expressed SLUG for 24 h (Figure S5C). The cells were then lysed, and ChIP was performed using anti-SLUG; ChIP primer 1 was used to detect SLUG binding with the* CLDN1* promoter. SLUG could bind the *CLDN1* promoter in 5-Aza-treated CL_1‑0_ cells, whereas no binding was detected in DMSO-treated control cells (Figure 5H). Taken together, these results indicated that DNA methylation might prevent SLUG recruitment to the *CLDN1* promoter and thereby abrogate SLUG-mediated transcriptional repression of* CLDN1*.

### TSA can facilitate *CLDN1* transcription by promoting histone modifications

In CL_1-5_ cells in which the *CLDN1* promoter was hypomethylated, TSA dramatically increased the levels of both CLDN1 RNA and protein (Figure 5I-J), and then the cells underwent the mesenchymal-epithelial transition (Figure S5D). We used ChIP assay to assess changes in histone modifications within the* CLDN1* promoter in CL_1-5_ cells in response to TSA; both activating (histone H3, lysine 4 trimethylation, H3K4me3) and repressive (H3K27me3) modifications were assayed. After treatment with TSA, H3K4me3 was enriched in the region containing the SLUG-binding site, whereas the prevalence of H3K27me3 was not altered (Figure 5K); no changes were evident within the region amplified by ChIP primer 2. The histone modification within the genes encoding glyceraldehyde-3-phosphate dehydrogenase (GAPDH) and hemoglobin subunit β (HBB) revealed that the change of histone modification for *CLDN1* was not global in the genome. (Figure S5E).

### RUNX3 upregulates *CLDN1* and represses *SLUG*

**Figure 6:**
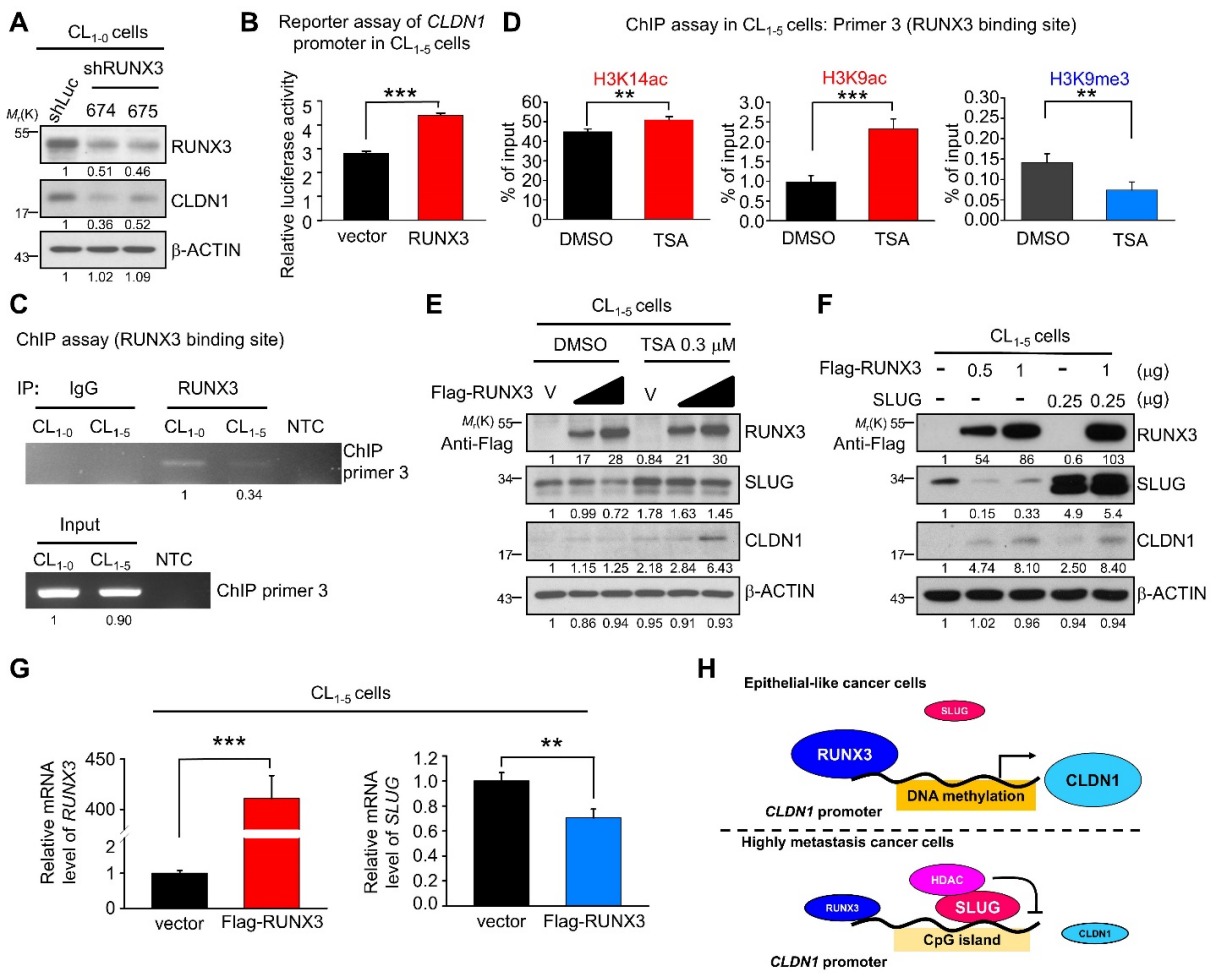
** RUNX3 upregulates *CLDN1* and downregulates *SLUG*. (A)** RUNX3 in CL_1-0_ cells was knocked down by lentivirus encoding two different shRNAs (shRUNX3 674 and 675) and the expression of RUNX3 and CLDN1 was observed by immunoblotting. **(B)** By the reporter assay, RUNX3 induced the promoter activity of *CLDN1*. **(C)** By the ChIP assay using the anti-RUNX3 antibody and the ChIP primer 3, endogenous RUNX3 bound to the *CLDN1* promoter. **(D)** After TSA treatment in CL_1-5_ cells, histone modification at the RUNX3-binding site of the *CLDN1* promoter was analyzed by the ChIP assay using anti-H3K14ac, anti-H3K9ac and anti-H3K9me. The enrichment of these histone marks was measured by RT-qPCR. **(E)** Ectopically RUNX3-overexpressing CL_1‑5_ cells were treated with TSA simultaneously for 6 h. The levels of SLUG and CLDN1 were observed by immunoblotting. **(F)** CL_1-5_ cells ectopically overexpressed RUNX3 and/or SLUG, and the expression of SLUG, RUNX3 and CLDN1 were detected by immunoblotting. **(G)** The mRNA expression of *SLUG* and *RUNX3* in RUNX3-overexpressing CL_1-5_ cells were detected by RT-qPCR.** (H)** The schematic illustration depicting the transcriptional regulation of *CLDN1* by RUNX3, SLUG, and epigenetics. The *n* values in **B** were three biologically independent experiments. The *n* values in **D** and** G** were three technical replicates per experiment. Error bars indicate in** B, D** and **G** represent the mean ± s.d. **p < 0.01, ***p < 0.001 in** B, D** and** G** (two-tailed Student's *t*-test). The input serves as the loading control and IgG serves as the specific binding control in the ChIP assay. β-ACTIN serves as the loading control in immunoblots.

Because we observed that the *CLDN1* transcription was up-regulation when the transcriptional repression ability of SLUG was inhibited by TSA (Figure 5I), we speculated that there are transcription activators contribute to the CLDN1 transcription. Based on analysis of the transcription factor binding site and searching of reference 23, RUNX3 was hypothesized to activate the expression of CLDN1 in lung cancer. Meanwhile, the level of RUNX3 was positively correlated with that of CLDN1 in lung cancer cells also implied that RUNX3 might involve in the regulation of CLDN1 expression. (Figure S5A). Therefore, we knocked down RUNX3 level in CL_1-0_ via a lentivirus encoding two different RUNX3-specific shRNAs, which resulted in the CLDN1 downregulation (Figure 6A). Subsequently, a reporter assay demonstrated that RUNX3 overexpression activated the *CLDN1*-promoter activity in CL_1-5_ cells (Figure 6B). Moreover, the ChIP assay (anti-RUNX3 antibody with ChIP primer 3, covering the RUNX3-binding site in the *CLDN1* promoter) revealed that endogenous RUNX3 could bind directly to the promoter and that the binding was greater in CL_1-0_ than in CL_1-5_ cells (Figure 6C).

Based on our finding that TSA promoted *CLDN1* transcription and altered the incidence of histone modifications at the SLUG-binding site of the *CLDN1* promoter in CL_1-5_ cells, we also assessed histone modifications within the RUNX3-binding site. ChIP revealed that activating marks H3K9ac and H3K14ac were enriched and the repressive mark H3K9me3 reduced within the RUNX3-binding site (Figure 6D). Furthermore, RUNX3 overexpression in CL_1-5_ cells induced CLDN1 level; when combined with TSA treatment, CLDN1 level was driven to even higher levels, indicating that other factors affected by TSA, such as SLUG, may contribute to CLDN1 protein expression (Figure 6E). Besides CLDN1 induction, ectopic expression of RUNX3 also suppressed endogenous SLUG expression (Figure 6E and 6F). To exclude the possibility that the observed RUNX3 overexpression-mediated upregulation of CLDN1 could be caused by SLUG downregulation, we ectopically overexpressed both RUNX3 and SLUG in CL_1-5_ cells, revealing that RUNX3 could induce CLDN1 expression even in cells overexpressing SLUG (Figure 6F). These results suggested that RUNX3 can directly bind the *CLDN1* promotor and drive *CLDN1* expression.

Finally, to explore how RUNX3 decreases SLUG level, CL_1-5_ cells were transfected with an empty vector or flag-RUNX3 plasmid and then treated with a proteasome inhibitor (MG132) or a protein synthesis inhibitor (cycloheximide). RUNX3 overexpression affected neither the proteasome-mediated degradation of SLUG (Figure S6A) nor SLUG stability (Figure S6B). Further, *RUNX3* overexpression decreased the level of *SLUG* mRNA (Figure 6G). Taken together, we demonstrated that *CLDN1* transcription could be activated by RUNX3 and repressed by SLUG as DNA hypomethylation (Figure 6H).

### Upregulation of *CLDN1* and *RUNX3* predict a positive chemotherapeutic response and clinical outcome for patients with lung adenocarcinoma

**Figure 7:**
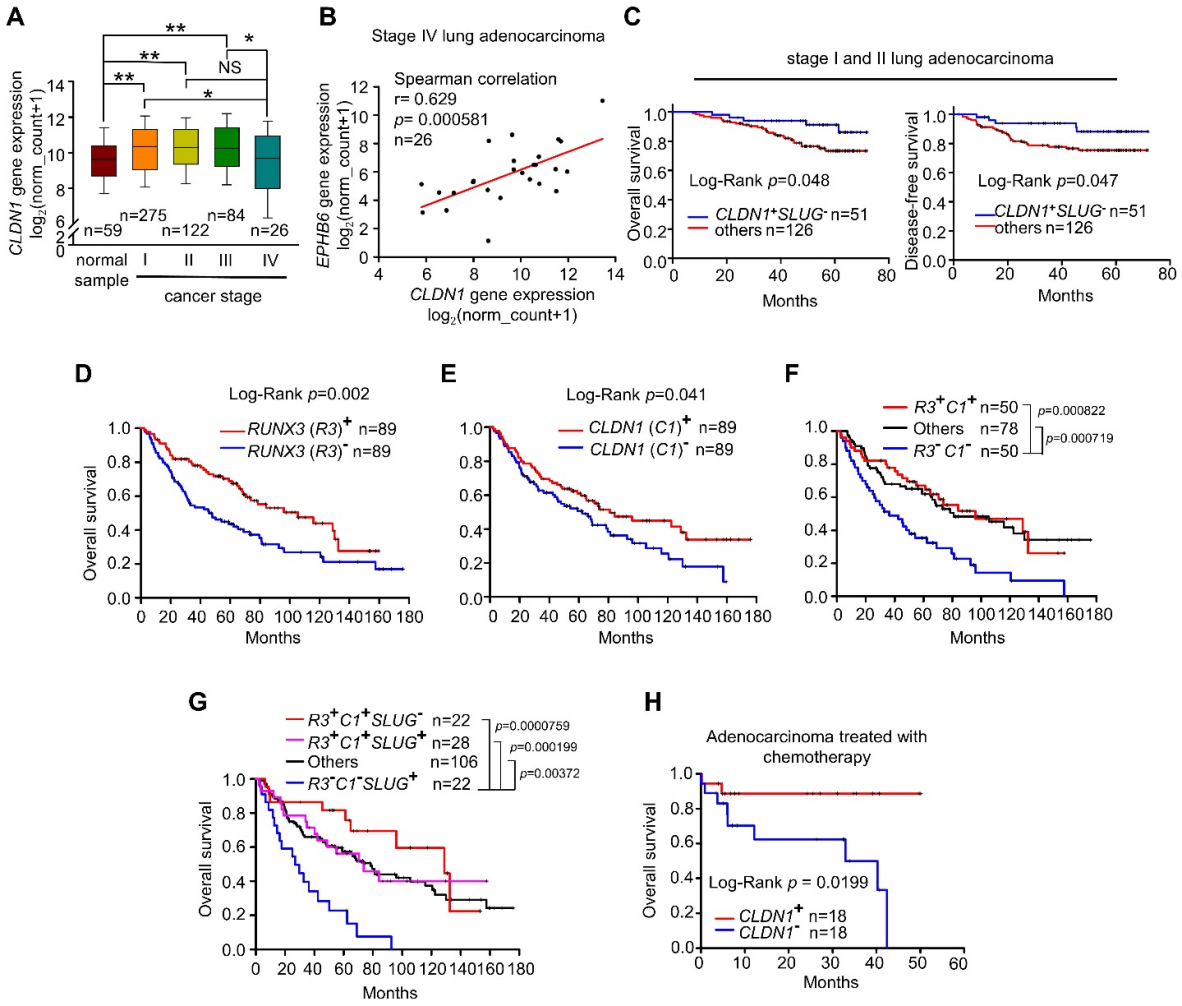
** Overexpression of *CLDN1* and *RUNX3* enhances the efficacy of chemotherapy and provides a survival benefit for patients with lung adenocarcinoma. (A)** Box-whisker plot showed the fluctuation of *CLDN1* expression through different tumor stages. The data were analyzed with one-way ANOVA, following post-hoc comparisons by Duncan's method. The data of the TCGA lung adenocarcinoma (LUAD) dataset were downloaded by the UCSC Xena website. *p < 0.05, **p < 0.01. **(B)** The correlation between *CLDN1* and *EPHB6* expression in stage IV lung adenocarcinoma (TCGA-LUAD) was estimated by Spearman correlation. **(C)** The overall survival and disease-free survival of the lung adenocarcinoma patients with elevated *CLDN1* expression but with low *SLUG* expression (*CLDN1*^+^*SLUG*^-^) and the other patients were estimated by the Kaplan-Meier plot. The data were downloaded from the GEO dataset, GSE31210. **(D-G)** The overall survival of the lung adenocarcinoma patients from GSE68465 (the cohort of the University of Michigan Cancer Center) was estimated by the Kaplan-Meier plots, based on expression levels of *RUNX3* (*R3*) **(D)**, *CLDN1* (*C1*)** (E)**, *R3 & C1* **(F)** or* R3, C1 & SLUG* (S2)** (G)**. **(H)** By the analysis from the Kaplan-Meier plotter website, *CLDN1* expression (*CLDN1*^+^) correlated with the overall survival of lung adenocarcinoma patients with chemotherapy., Lung cancer patients in C‑H were classified into high and low expression groups for specific genes (*RUNX3, CLDN1,* or *SLUG*), using the median expression of genes as the cut-off value. The *n* values in A-H were indicated in each image. The *p* values in C-E and H were determined by the log-rank test. The *p* values in F and G were determined by log-rank test, following pairwise multiple comparisons by Holm-Sidak method.

Lung adenocarcinoma data obtained from The Cancer Genome Atlas (TCGA) indicates that *CLDN1* expression differs between tumor samples and normal samples. In samples from patients with early-stage lung adenocarcinoma, *CLDN1* expression was elevated compared with normal samples (Figure 7A). Similar results were observed in GEO dataset, GSE27262, that was collected from samples from patients with stage I adenocarcinoma (Figure S7A). Importantly, *CLDN1* expression was downregulated (Figure 7A) and correlated with the downregulated expression of *EPHB6* (Figure 7B) in stage IV lung adenocarcinoma, suggesting that *CLDN1* may correlate with *EPHB6* expression and be a metastasis suppressor. Based on our results, the CLDN1-SLUG axis repressed lung adenocarcinoma progression, we investigated whether patients with an intact CLDN1-SLUG axis lived longer. We analyzed the GEO dataset GSE31210 that was collected from stage I and II lung adenocarcinomas. The patients with elevated *CLDN1* expression but with low *SLUG* expression had longer overall survival and longer disease-free survival than other adenocarcinoma patients (Figure 7C).

Next, we investigated whether there is a clinical connection between *CLDN1* and *RUNX3* expression with respect to the survival of patients with lung adenocarcinoma. Indeed, upregulated expression of either* RUNX3* or *CLDN1* was a good prognostic marker for overall survival (Figure 7D-E). Interestingly, patients with high levels of both* CLDN1* and* RUNX3* had better overall survival than those with low levels of these proteins (*P* = 0.000822) (Figure 7F)**.** Importantly, patients with downregulated *SLUG* accompanied by high levels of *CLDN1* and *RUNX3* (R3^+^C1^+^S2^-^) had better overall survival than patients with upregulated *SLUG* accompanied by low levels of *CLDN1* and *RUNX3* (R3^-^C1^-^S2^+^) (*P* = 0.0000759) (Figure 7G). We also found that upregulation of both RUNX3 and CLDN1 enhanced the sensitivity of cancer cells to cisplatin and induced cell death (Figure S7B). Therefore, CLDN1 and RUNX3 are good prognostic markers for lung adenocarcinoma.

### Histone deacetylase inhibitors and cisplatin had a synergistic cytotoxic effect on CLDN1^low^ cancer cells

**Figure 8:**
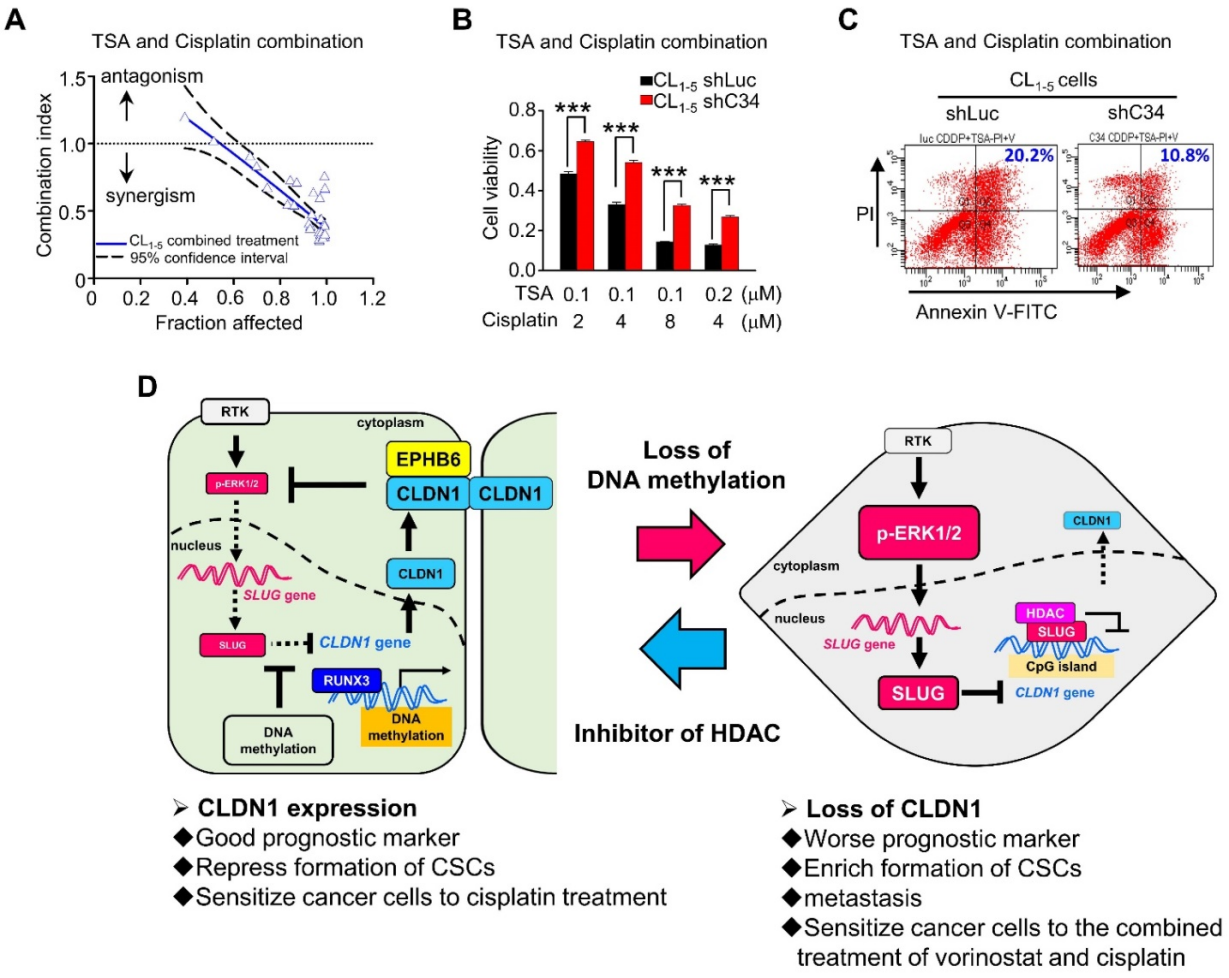
** Histone inhibitors and cisplatin had a synergistic cytotoxic effect. (A)** Upon combined treatment with TSA and cisplatin, it showed the synergistic effect on CL_1-5_ cells which expressed the low level of *CLDN1*. **(B)** CLDN1 knockdown in CL_1-5_ cells increased the cell viability under treatments with combined different ratios of TSA and cisplatin.** (C)** By the annexin V/PI assay, CLDN1 knockdown decreased the percentage of cell death (Q2 + Q4) induced by combined treatment with TSA and cisplatin.** (D)** The schematic illustration of CLDN1-mediated suppression of cancer progression and the regulation of *CLDN1* transcription are shown. CLDN1 inhibits ERK1/2 signaling through EPHB6 and leads to SLUG downregulation. SLUG downregulation plays a crucial role in the suppression of cancer progression. Interestingly, hypermethylation within the SLUG-binding site of the *CLDN1* promoter can prevent SLUG binding and maintain *CLDN1* transcription when RUNX3 directly drives *CLDN1* transcription. Thus, a reciprocal regulation exists between CLDN1 and SLUG. Besides, when CLDN1 is deficient, SLUG would up-regulate and bind the *CLDN1* promoter to further repress CLDN1. Finally, CLDN1 protein sensitizes cancer cells to cisplatin, and represses the formation of CSCs, and is a good prognostic marker. HDAC inhibitor TSA and cisplatin would have a synergistic effect on cancer cell death because of CLDN1 expression. The *n* values in **A** and** B** were three biologically independent experiments. Error bars indicated in** B** represent the mean ± s.e.m. ***p < 0.001 (two-tailed Student's *t*-test).

Based on our studies, CLDN1 protein level sensitizes cancer cells to chemotherapy drugs. To confirm that *CLDN1* expression is a positive predictor for patients who respond well to chemotherapy, we analyzed the clinical correlation between *CLDN1* expression and survival of lung adenocarcinoma patients who had received chemotherapy using the KM plotter website. Patients with the *CLDN1*^high^ phenotype had significantly longer survival than patients with the *CLDN1*^low^ phenotype (*P* = 0.0199) (Figure 7H). Given that TSA activated *CLDN1* expression in CLDN1^low^ lung adenocarcinoma cells (Figure 5I and 5J) and CLDN1^high^ cells were more sensitive to cisplatin (Figure 3H-L and 4D-G), TSA may promote cisplatin-induced cell death in *CLDN1*^low^ cancer cells. We therefore treated CL_1-5_ cells with a combination of cisplatin and TSA (Figure S8A). Cell viability was used to calculate the combination index. All the combination index values were smaller than 1 at different “fraction affected” values (representing the proportion of dead cells) (Figure 8A). This indicated that TSA combined with cisplatin had a synergistic effect on CL_1-5_ cell death. To explore whether this synergistic effect was the result of CLDN1 induction, we established a CLDN1-knockdown in CL_1-5_ cells (CL_1‑5_/shC34) for testing the effect of TSA. Cell viability was measured for CL_1‑5_/shC34 cells after combined treatment with TSA and cisplatin. The combined treatment had more cytotoxic to CL_1-5_/shLuc than CL_1‑5_/shC34 cells (Figure 8B). Similarly, annexin V/PI staining revealed more cell death among CL_1-5_/shLuc than CL_1-5_/shC34 cells (representative image in Figure 8C and quantification in Figure S8B). Moreover, Hs68 (immortalized fibroblasts) did not respond to the drug combination (Figure S8C). We further used vorinostat (FDA-approved HDAC inhibitor) to confirm the combined effect. Similarly, vorinostat combined with cisplatin had a synergistic effect on CL_1-5_ and was more cytotoxic to CL_1-5_/shLuc than CL_1-5_/shC34 cells (Figure S8D and S8E). These results suggested that CLDN1 may be a useful prognostic predictor of chemotherapeutic efficacy for lung adenocarcinoma patients. Thus, forced CLDN1 expression may improve the survival of low CLDN1-expressing patients who undergo treatment with cisplatin.

This study (Figure 8D) revealed that CLDN1 inhibits ERK1/2 signaling through EPHB6 and leads to SLUG downregulation. SLUG downregulation plays a crucial role in the suppression of cancer progression, including cell mobility, the formation of CSCs, and drug resistance. Interestingly, hypermethylation within the SLUG-binding site of the *CLDN1* promoter can prevent SLUG binding and maintain* CLDN1* transcription. Additionally, one transcription activator, RUNX3, can directly bind the *CLDN1* promoter and drive *CLDN1* transcription, leading to suppression of cancer progression. When CLDN1 is downregulated, SLUG is activated and binds the *CLDN1* promoter, resulting in further downregulation of CLDN1 and the promotion of cancer progression. Thus, a reciprocal regulation exists between CLDN1 and SLUG. Taken together, we decipher the regulation of CLDN1 and uncovers its ability to repress cancer stemness and sensitizes chemotherapy. CLDN1 is a stratification biomarker to group CLDN1^high^ and CLDN1^low^ patients who will get a different response to chemotherapy. We suggest that CLDN1^low^ patients will get a better response to the combined treatment of chemotherapy and vorinostat.

## Discussion

Recent studies revealed that DNA methylation suppresses gene expression 55. In contrast, our results suggest that DNA methylation prevents SLUG recruitment to the *CLDN1* promoter, thus maintaining *CLDN1* expression. Therefore, the epigenetic status of the epithelial genes modulates the EMT. This may explain why sometimes the plasticity of the EMT does not change when cells are exposed to TGF-β or express EMT regulators.

*CLDN1* is a direct target of SLUG 54. Here, we describe the CLDN1-EPHB6-ERK1/2-SLUG axis, in which CLDN1 has an important role in repressing SLUG expression to inhibit cell migration. This reciprocal negative-feedback loop explains why cell migration decreases as tight junctions form and increases if cell boundaries become disrupted. This regulation may represent the normal balance of epithelial-cell dynamics, which is lost in cancer. The switch in the reciprocal regulation of CLDN1 and SLUG might be important for suppressing metastasis and the formation of CSCs.

Our results revealed that CLDN1 is a metastasis suppressor in lung adenocarcinoma. Previous studies have shown that the CLDN1 involvement in suppressing or activating metastasis is paradoxical among different cancers 56. This apparent discrepancy may be attributable to the differential subcellular localization of CLDN1. One study suggested that TNFα-induced cell migration is through cytoplasmic CLDN1 in lung carcinoma cells 57. Upon loss of normal cell boundaries, membrane-bound CLDN1 translocates to the cytoplasm, and cytoplasmic CLDN1 promotes cell migration. Similarly, cytoplasmic CLDN1 promotes cell migration in metastatic melanoma 25. By contrast, in nevi and less aggressive melanomas, CLDN1 predominantly localizes in the nucleus or cell junctions 25 and the nuclear-localized CLDN1 mutant (S69A) does not increase cell invasion 58. Therefore, it is the membrane-bound form of CLDN1 that suppresses metastasis. Moreover, only the membrane form of CLDN1 induces apoptosis in breast-tumor spheroids 59. Consistent with these results, our data demonstrate that collaboration between CLDN1 and EPHB6 at the cell membrane suppresses SLUG expression in lung adenocarcinoma cells, which inhibits the formation of CSCs and decreases cell migration capacity.

Our results demonstrate that CLDN1 inhibits ERK1/2 signaling through EPHB6, resulting in suppression of SLUG expression; EFNB2, an EPHB6 ligand, inhibited cell migration only in CLDN1-overexpressing cells. EPHB6 is a strong metastasis suppressor 31 and has a biphasic function in cell migration, i.e., EPHB6 promotes migration under low-dose stimulation with EFNB2, whereas it suppresses migration under high-dose stimulation 60. Moreover, EPHB6 can be a positive 61 or negative 62 regulator of ERK signaling, and this might be attributable to the activation status of EPHB6. Moreover, CLDN1 can interact with and activate another EPHB6 ligand, EFNB1, which promotes cell-cell adhesion 63. Accordingly, we suggest that CLDN1 may recruit EPHB6 and EFNB1/2 at the cell membrane, thereby enhancing the activation of EPHB6 to suppress cell migration. Therefore, CLDN1 appears to regulate the function of EPHB-ephrin family members.

According to our results, CLDN1 is upregulated by vorinostat treatment as well as RUNX3 overexpression, suggesting that restoration of RUNX3 may be another strategy to upregulate the CLDN1 expression. Interestingly, Studies show pan‑HDAC inhibitor, such as TSA, could be the restoration of RUNX3 by stabilizing the protein expression and enhance the transcriptional activity of RUNX3 64, 65. Therefore, HDAC inhibitors could increase CLDN1 expression by suppressing SLUG activity and/or restoration of RUNX3. RUNX3 is a potent tumor suppressor in gastric cancer 66 and we demonstrated that lung cancer patients harboring the RUNX3-CLDN1 axis have better overall survival, suggesting RUNX3 may be a tumor suppressor in lung cancer. Indeed, loss of RUNX3 is an early event in lung adenocarcinoma 67, suggesting the RUNX3-CLDN1 axis plays an essential role in repressing lung cancer progression.

Both TCGA data and the GEO dataset show that *CLDN1* is upregulated in stage I and II lung adenocarcinoma but downregulated in stage IV lung adenocarcinoma. Notably, no genetic alteration has been identified in *CLDN1* in cancer 68. We explored the transcriptional regulation of *CLDN1*, including any effects of epigenetic modifications and the activation of the transcriptional activator RUNX3 and transcriptional repressor SLUG. *CLDN1* upregulation early during lung tumorigenesis may be caused by methylation of the *CLDN1* promoter, as illustrated in our study, whereas its decrease in stage IV tumors may be mediated by a shift in gene activation, i.e., to *SLUG*. Additionally, our data show that *CLDN1* is a predictive marker for chemotherapy response. Lung adenocarcinoma patients with *CLDN1* expression live longer, which may be a result of metastasis suppression and/or sensitization of cancer cells to chemotherapy. Indeed, we found that HDAC inhibitors could reactivate *CLDN1* expression in tumor cells, and combined treatment with cisplatin and TSA or vorinostat had a synergistic cytotoxic effect. However, phase I/II clinical studies have shown that vorinostat alone provides no benefit to patients with non-small-cell lung cancer 69, yet it enhances the efficacy of carboplatin and paclitaxel in advanced cases of non-small-cell lung cancer—although the apparent improvements in progression-free survival and overall survival have not reached statistical significance 70, 71. Based on our current results, lung adenocarcinoma patients with the CLDN1^low^ phenotype may benefit from combined treatment with vorinostat and chemotherapy. Forced CLDN1 expression in low CLDN1-expressing lung adenocarcinoma will increase the chemotherapy response, providing a novel therapeutic strategy.

## Supplementary Material

Supplementary figures and tables.

## Figures and Tables

**Table 1:** To estimate the frequency of CSCs by extreme limiting dilution analysis.

Tumor xenograft rate				
Hop62 cell number	shL-L	shL-C34	shS3-L	shS3-C34
5x10^5^	6/6	6/6	6/6	6/6
5x10^4^	6/6	6/6	5/6	4/6
5x10^3^	5/6	6/6	2/6	3/6
5x10^2^	2/6	6/8	0/6	1/6
stem cell frequency	1/2239	1/361	1/22951	1/24080
Pairwise tests *p* value		** 0.00453 (vs shL-L)		NS 0.934 (vs shS3-L)

The *n* values per group are indicated in each group. The significance of apparent differences in values from pairwise tests was determined by the chi-squared test. shL-L: shLuc-shLuc; shL-C34: shLuc-shC34; shS3-L: shSLUG-S3-shLuc; shS3‑C34: shSLUG-S3-shC34.

## Abbreviations

5-Aza: 5-Azacytidine
ALDH1A1: aldehyde dehydrogenase (ALDH) 1 family member A1
ChIP: chromatin immunoprecipitation
CHX: cycloheximide
CLDN1: claudin 1
CSCs: cancer stem-like cells
DEAB: diethylaminobenzaldehyde
EFNB2: ephrin B2
EMT: epithelial-mesenchymal transition
EPHB6: ephrin receptor B6
GEO: gene expression omnibus
HA: hemagglutinin
H3K4me3: histone H3, lysine 4 trimethylation
H3K27me3: histone H3, lysine 27 trimethylation
H3K9ac: histone H3 lysine 9 acetylation
H3K14ac: histone H3 lysine 14 acetylation
H3K9me3: histone H3 lysine 9 trimethylation
HDACs: histone deacetylases
IF: immunofluorescence
IP: immunoprecipitation
KD: knocked down
LUAD: lung adenocarcinoma
MAPK: mitogen-activated protein kinase
NS: non-significant
O/E: overexpression
RT-qPCR: reverse-transcription quantitative PCR
RUNX3: RUNX family transcription factor 3
shRNAs: short hairpin RNAs
TCGA: the cancer genome atlas
TSA: trichostatin A
