# Supplementary material for: DNA methylation maintains the CLDN1-EPHB6-SLUG axis to enhance chemotherapeutic efficacy and inhibit lung cancer progression
Source: Theranostics. 2020 Jul 11;10(19):8903–23. doi: 10.7150/thno.45785 (PMC7392003; doi:10.7150/thno.45785)
Supplement: Supplementary file 1 — Supplementary figures and tables. [file thnov10p8903s1.pdf]

Supplementary Figure legends

Supplementary Figure S1. CLDN1 regulates cell mobility by repressing SLUG expression through the ERK1/2 pathway. (**A**) cDNA microarray data (GSE10309) for CL_{1-5} cells (overexpressing CLDN1) were used to analyze signaling pathways using the bioinformatics tool, MetaCore. (**B**) The basal levels of CLDN1 in lung cancer cell lines were detected by RT-qPCR (three technical replicates per experiment). Error bars represent the mean ± s.d. $^{**}p < 0.01$, $^{***}p < 0.001$ (two-tailed Student's *t*-test). (**C**) CL_{1-5} cells were transfected with empty vector or CLDN1 plasmids. The cells were starved for 8 h and then changed into the complete medium for the indicated time. The expressions of CLDN1, phosphorylated or total ERK1/2, and β-ACTIN were assessed by immunoblotting. (**D**) Immunoblotting for CLDN1, phosphorylated or total ERK1/2, and β-ACTIN in Hop62 cells with CLDN1 silence starved for 2 h or not. (**E**) Hop62 cells were infected with lentivirus-based shRNA targeting LacZ or CLDN1. The shC33 and shC34 represent different shRNAs of CLDN1. The cells were starved for 8 h and then changed into the complete medium (containing 10% FBS) for the indicated time. The expressions of CLDN1, phosphorylated or total JNK, and β-ACTIN were assessed by immunoblotting. **(F)** The

expressions of these EMT-TFs in CLDN1-silenced cells were checked by RT-qPCR (three technical replicates per experiment). Error bars represent the mean ± s.d. ***p* < 0.01, ****p* < 0.001 (two-tailed Student’s *t*-test). NS: non-significant. **(G)** The expressions of these EMT-TFs in CLDN1-overexpressed cells were checked by Western blot. β-ACTIN serves as the loading control in immunoblots. The samples were the same as Figure 1A and performed Western blot at the same time. (**H**) CL_{1-5} was starved for 24 h and then treated 20 μM PD98059 for 30 min. The expressions of SLUG, phosphorylated or total ERK1/2, and β-ACTIN were assessed by immunoblotting. (**I**) The SLUG was knockdown by lentivirus-based shRNA in the cells with CLDN1 silence. The expression of CLDN1, SLUG, E-cad and β-ACTIN was assessed by immunoblotting. β-ACTIN serves as the loading control in immunoblots. (**J**) Hop62 cells with knockdown of CLDN1 and/or SLUG subcutaneously (shLuc-shLuc, shC34-shLuc, shLuc-shSLUG-S3, and shC34-shSLUG-S3) inoculated in NOD-SCID mice, and lung cancer metastasis was observed for six months (*n* = 3 or 4 mice per group). The arrowhead indicates lung metastasis.

Supplementary Figure S2. EPHB6 is upregulated upon CLDN1 overexpression as assessed with cDNA microarrays. (**A**) The receptor

tyrosine kinases (RTKs), which suppress distance metastasis were analyzed in our cDNA microarray data. The expression level of RTK in CL_{1-5} cells overexpressing CLDN1 was compared with vector control and got the fold change. The values in the parentheses represent the number of different probes for one gene. The asterisk indicates that only EPHB6 was highly expressed upon the overexpression of CLDN1. (**B**) The phosphorylation of ERK1/2 was measured by immunoblotting in Hop62 cells and CLDN1 silence cells when ephrin B2 treatment.

Supplementary Figure S3. CLDN1 represses cancer stemness and sensitizes lung adenocarcinoma cells to chemotherapeutic drugs *in vitro*. **(A)** The correlation between the stemness score and *CLDN1* expression was analyzed by Pearson correlation in the TCGA-LUAD cohort. (**B**) The mRNA level of *SLUG* was analyzed by RT-qPCR as CLDN1-knockdown Hop62 cells were incubated in monolayer or sphere condition. (**C** and **D**) The quantified data of Figures 3C and 3D are shown here. ALDH activity was measured by Aldefluor assay in the cells with CLDN1 knockdown or overexpression. (**E**) The knockdown efficiency of CLDN1 was measured by RT-qPCR (three technical replicates per experiment). (**F**) The cytotoxicity of carboplatin and taxol was

measured in CLDN1-knockdown Hop62 cells, and the IC_{50} was calculated by CalcuSyn software. (**G**) The cytotoxicity of carboplatin and taxol was measured in CLDN1-overexpressing CL_{1-5} cells, and the IC_{50} was calculated by CalcuSyn software. (**H**) The percentage of cell death in figure 3J was shown here. The annexin V/PI assay evaluated the percentage of cell death as CLDN1-overexpressing CL_{1-5} cells were treated with cisplatin. (**I**) The cell cycle analysis of CL_{1-5} cells overexpressing CLDN1 or vector was analyzed by PI staining using flow cytometry. The representative image (left) and quantification of the percentage of cell-cycle phases (right) are shown. The *n* values in **B, C, D, F, G** and **H** were three biologically independent experiments. Error bars indicated in **B, C, D, E** and **H** represent the mean ± s.d. Error bars indicated in **F** and **G** represent the mean ± s.e.m, $^{**}p < 0.01$, $^{***}p < 0.001$ (two-tailed Student's *t*-test).

Supplementary Figure S4. CLDN1 represses cell proliferation and sensitizes lung adenocarcinoma to cisplatin *in vivo.* (**A**) The cell proliferation of vector- (p1511) or CLDN1-overexpressing CL_{1-5} (pc1513 and pc1515) was measured. (**B**) The representative image (left) and the quantification (right) of anchorage-independent growth of p1511, pc1513 and

pc1515 by the soft agar assay are shown. The colonies (the diameter is over 100 μm) were counted. (**C-F**) The tumor-bearing mice received cisplatin to evaluate the sensitivity of CLDN1-overexpressing cancer cells (pc1515) to cisplatin. The schedule of cisplatin treatment and tumor mass (**C**), tumor volume (**D**), tumor weight (**E**), and body weight (**F**) of the tumor-bearing mice are shown (n = 8 mice per group). The n values in **A** and **B** were three biologically independent experiments. Error bars indicated in **A, B, D** and **F** represent the mean ± s.d., **p < 0.01 (two-tailed Student's t-test).

Supplementary Figure S5. DNA hypermethylation of the *CLDN1* promoter maintains its transcription by abrogating SLUG-mediated suppression. (**A**) Immunoblotting showed the protein expression of CLDN1-EPHB6-ERK1/2-SLUG axis between $CL_{1\text{-}0}$ and $CL_{1\text{-}5}$. (**B**) The results of bisulfite sequencing showed the methylation patterns of the *CLDN1* promoter in $CL_{1\text{-}0}$ and $CL_{1\text{-}5}$ (top). The CpG island of the *CLDN1* promoter was predicted by the MethPrimer website (bottom). Shown are regions of pyrosequencing or methylation-specific PCR. (**C**) Immuno-blotting showed the ectopic overexpression of SLUG in $CL_{1\text{-}5}$. (**D**) The cell morphology of $CL_{1\text{-}5}$ cells treated with TSA showed the mesenchymal-epithelial transition. (**E**) $CL_{1\text{-}5}$ cells were treated with TSA and

then the ChIP assay was performed using H3K4me3 or H3K27me3 antibodies and primers which amplified their positive controls, glyceraldehyde-3-phosphate dehydrogenase (GAPDH) or hemoglobin beta subunit, respectively (HBB).

Supplementary Figure S6. Ectopic flag-RUNX3 does not influence the degradation of SLUG. (**A**) The CL_{1-5} cells ectopically overexpressed RUNX3 and were treated with MG132, the proteasome inhibitor. SLUG expression was observed by immunoblotting (left) and the density of bands of SLUG was quantified and normalized to each vector group in the DMSO or MG132 treatments (right). The *n* values were two biologically independent experiments. NS: non-significant (two-tailed Student's *t*-test). Error bars represent the mean ± s.d. (**B**) The CL_{1-5} cells ectopically overexpressed RUNX3 and were treated with the protein synthesis inhibitor cycloheximide (CHX) at different times. The SLUG protein levels were observed by immunoblotting and quantified the density of band of SLUG at different time points. The experiment was performed a single time.

Supplementary Figure S7. Overexpression of *CLDN1* and *RUNX3* enhances the efficacy of chemotherapy and provides a survival benefit for patients with lung adenocarcinoma. (**A**) GSE 27262 dataset showed *CLDN1* expression between normal-tumor paired samples. (**B**) CL_{1-5} cells overexpressed RUNX3 and CLDN1 and then were treated with 10 μM cisplatin for 8 h. The cleaved PARP was observed by immunoblotting and defined as apoptosis. The *n* values in **A** were shown in each image. ***$p < 0.001$ (two-tailed Student's *t*-test).

Supplementary Figure S8. Histone inhibitors and cisplatin had a synergistic cytotoxic effect on CLDN1^{low} cancer cells.

(**A**) CL_{1-5} cells were treated with the serial concentration of TSA which combined with three concentrations of cisplatin (CDDP) (left) or conversely, treated with the serial concentration of cisplatin which combined with three concentrations of TSA (right). The cell viability was measured by WST-1 and normalized to the cells with no drug treatment. (**B**) The cell death (Q2 + Q4) of figure 7K was quantified. *n* = two biologically independent experiments**.** Error bars represent the mean ± s.d. (**C**) The cell viability of Hs68 cells was treated with a combination of TSA and cisplatin. (**D**) The cell viability of CL_{1-5} cells receiving

combined treatment of vorinostat (SAHA) and cisplatin was used to calculate the combination index and exhibited the synergistic effect. (**E**) CLDN1 knockdown in CL_{1-5} cells increased the cell viability under treatments with combined different ratios of SAHA and cisplatin. The *n* values in **A, C, D** and **E** were three biologically independent experiments. Error bars indicated in **A, C** and **E** represent the mean ± s.e.m. $^{**}p < 0.05$, $^{***}p < 0.001$ in **B,** and **E** (two-tailed Student's *t*-test).

Supplementary Figure S1.

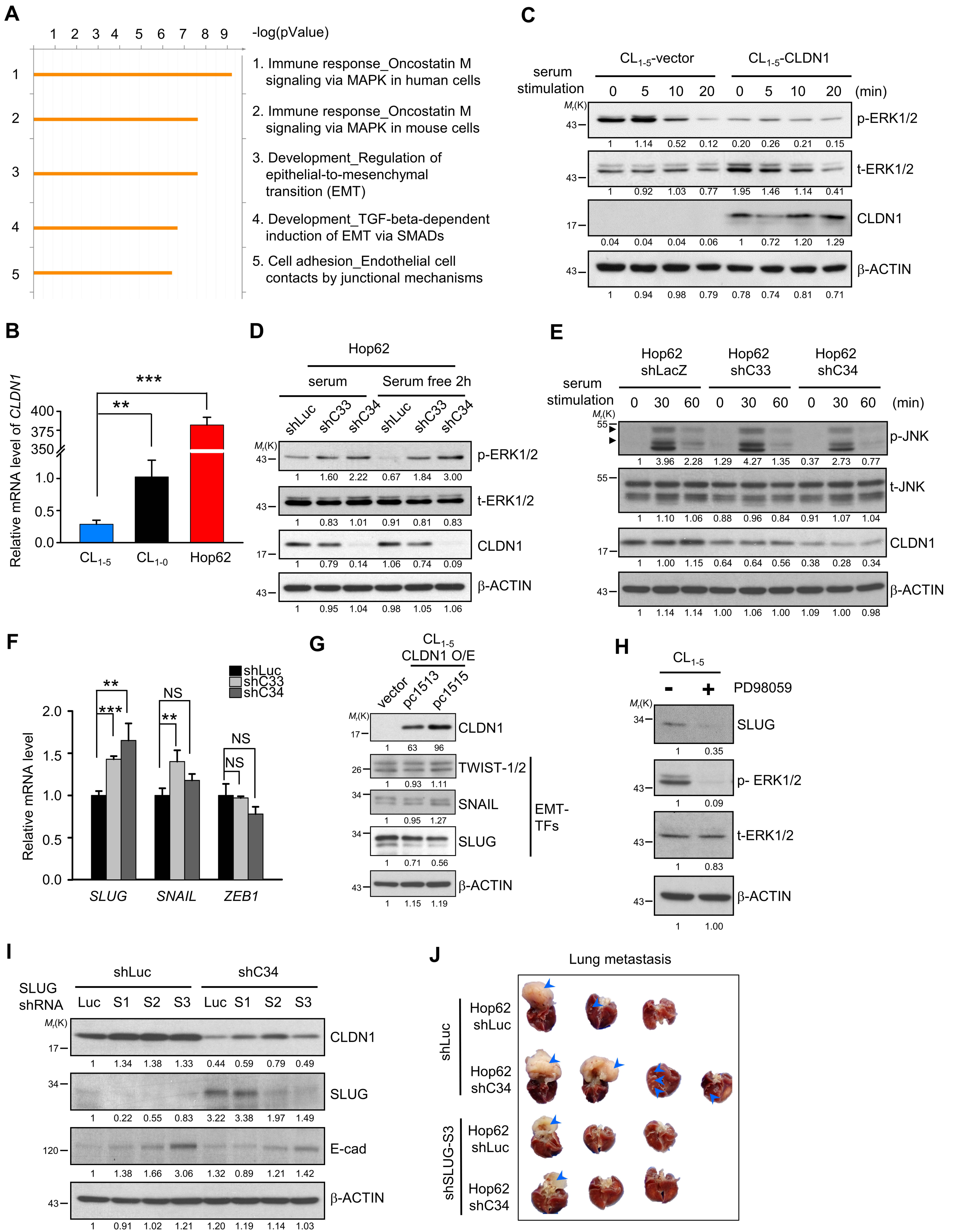

Supplementary Figure S2.

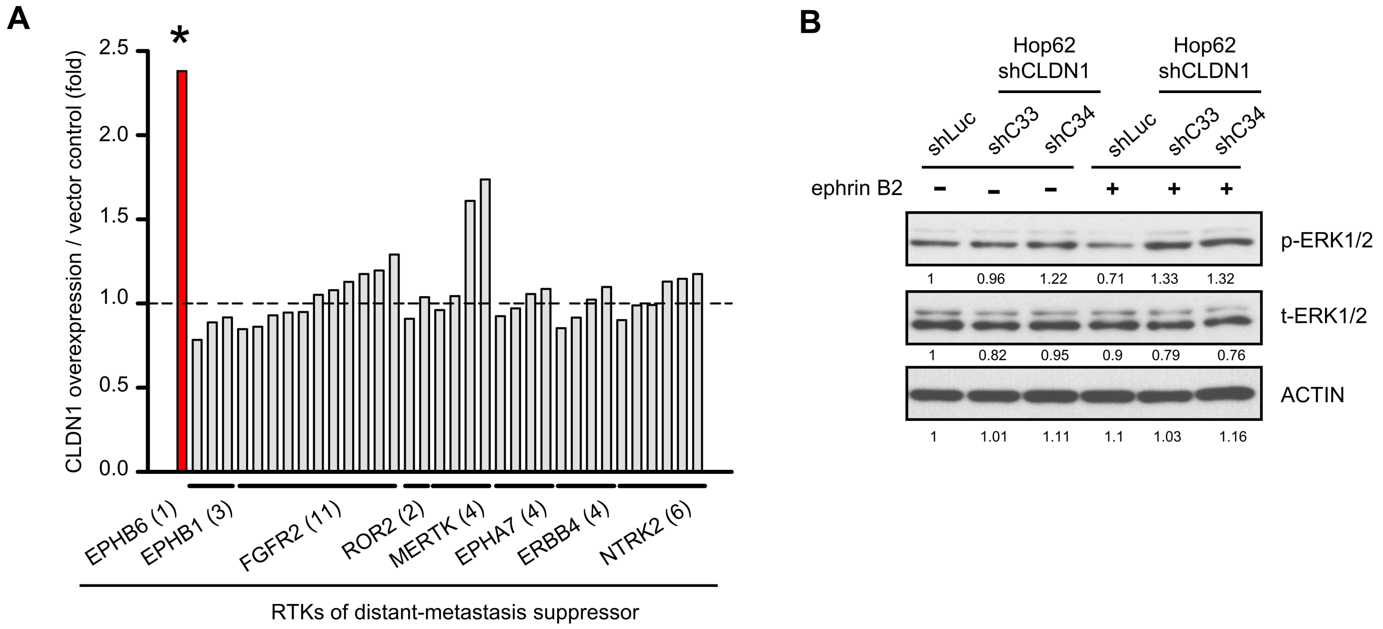

Supplementary Figure S3.

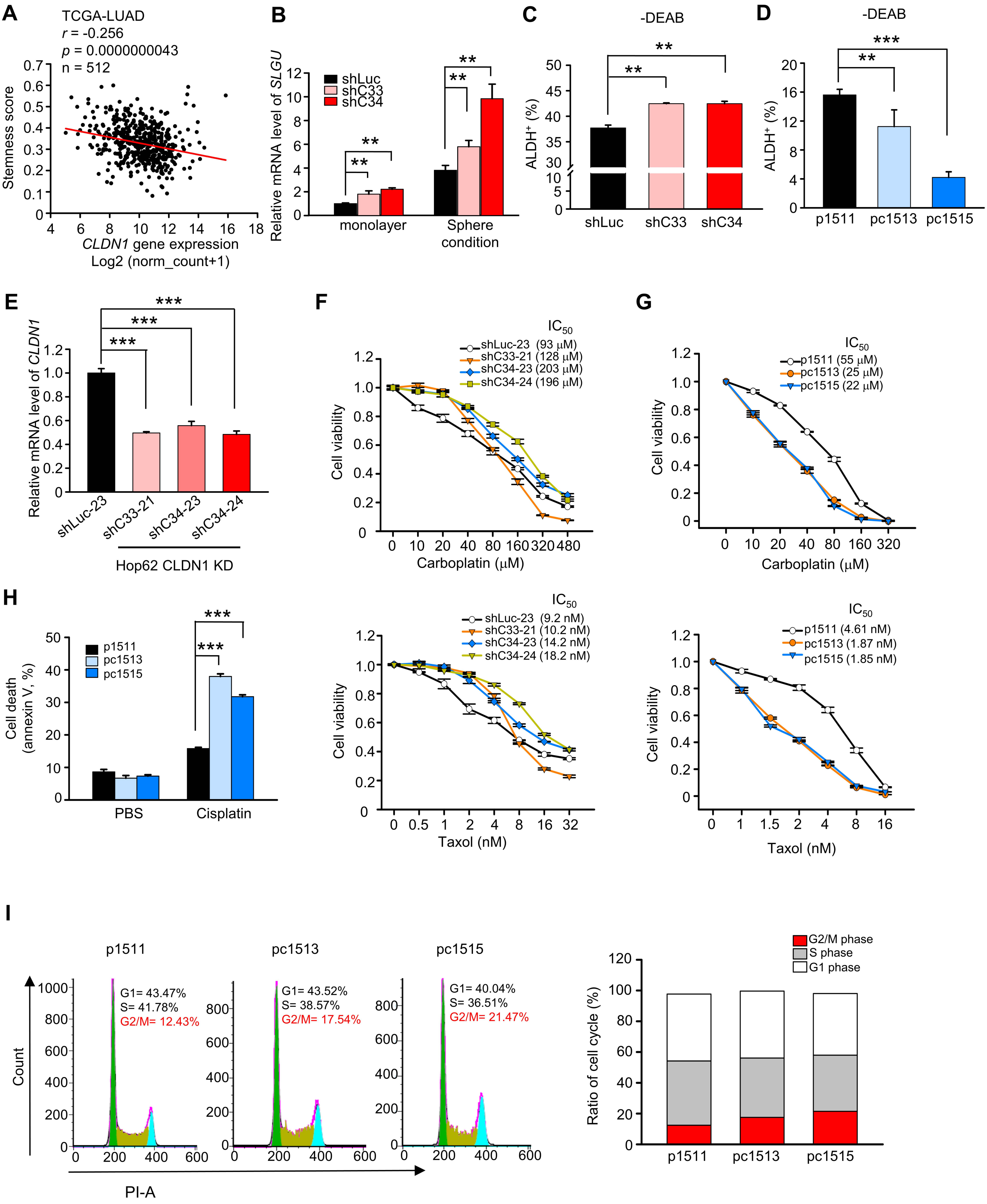

Supplementary Figure S4.

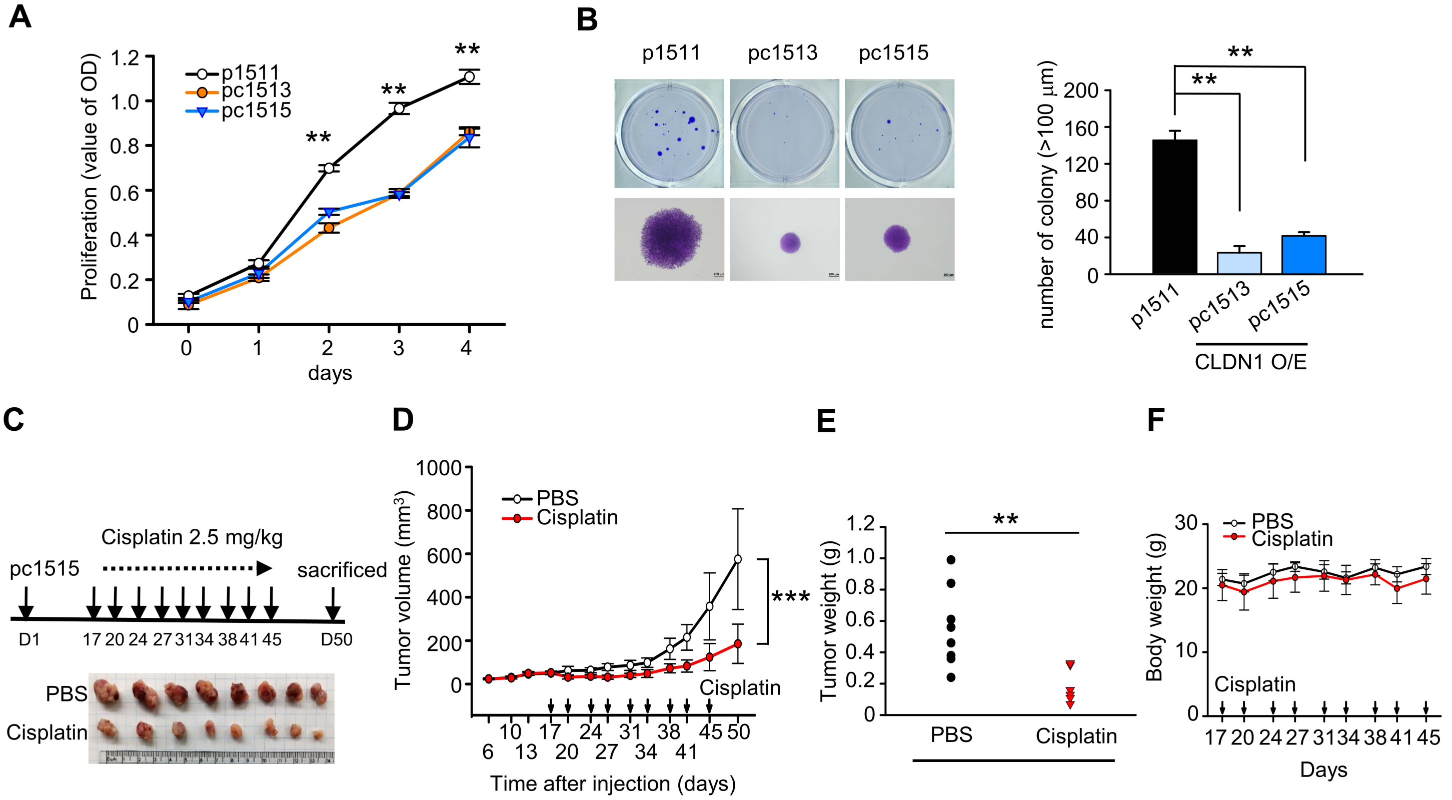

Supplementary Figure S5.

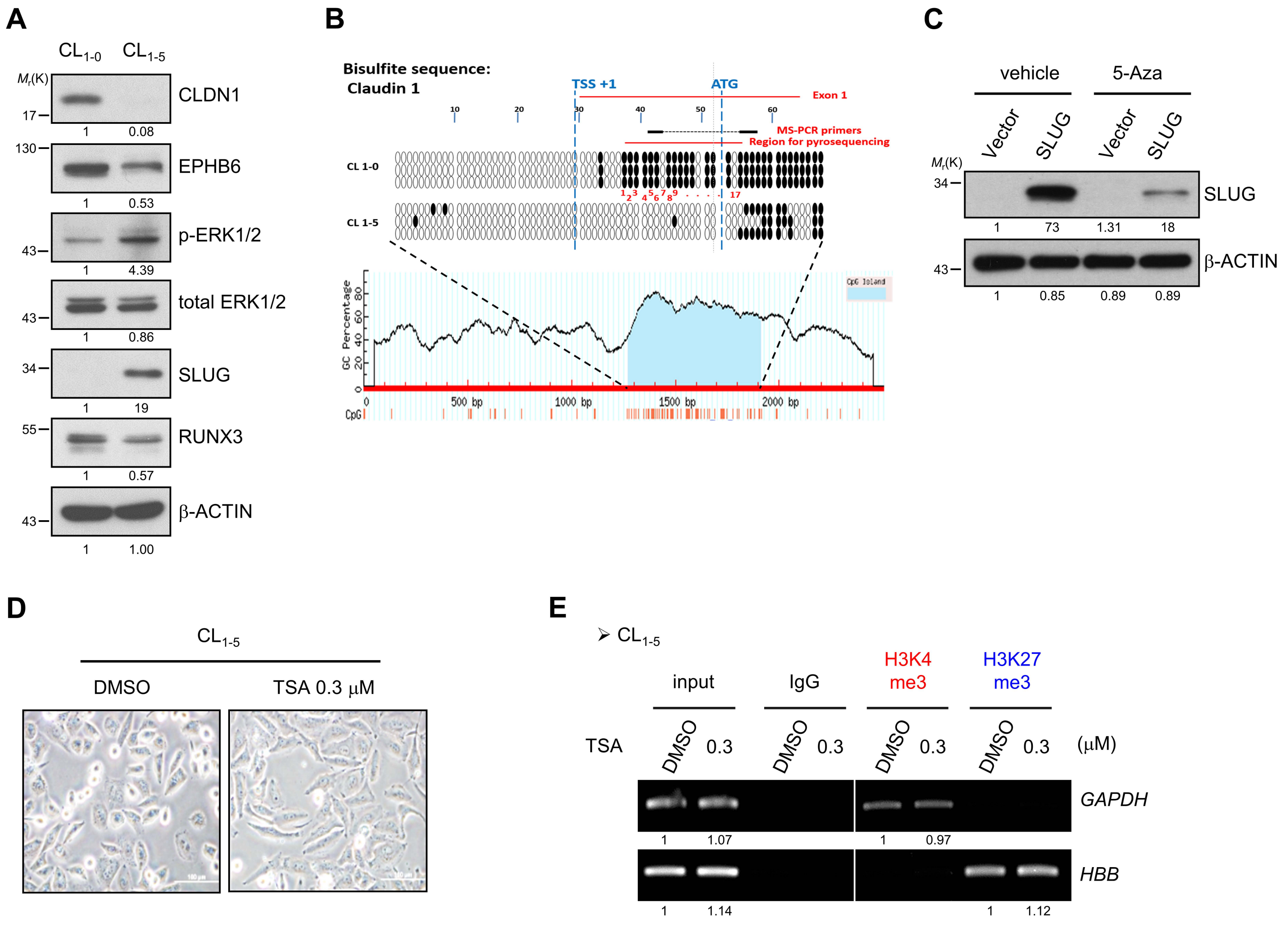

Supplementary Figure S6.

A

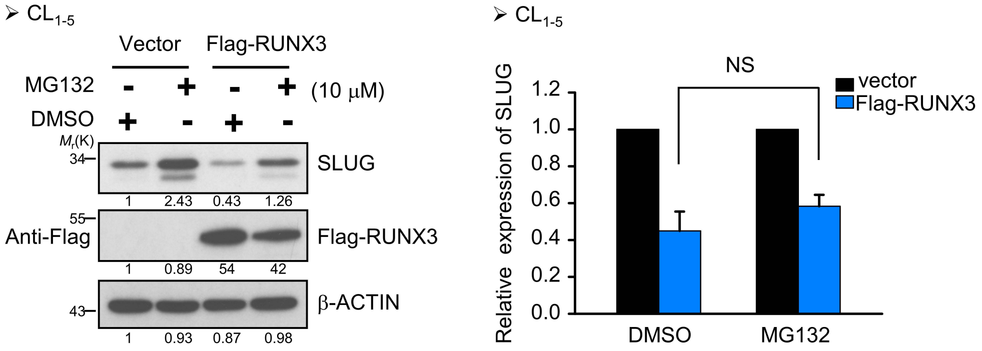

B

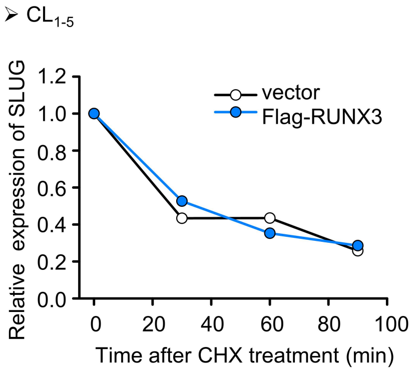

Supplementary Figure S7.

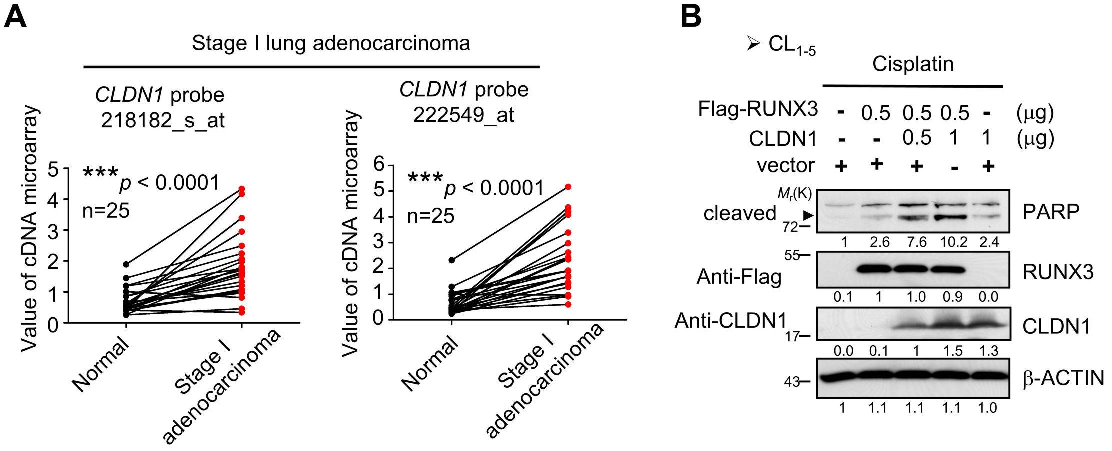

Supplementary Figure S8.

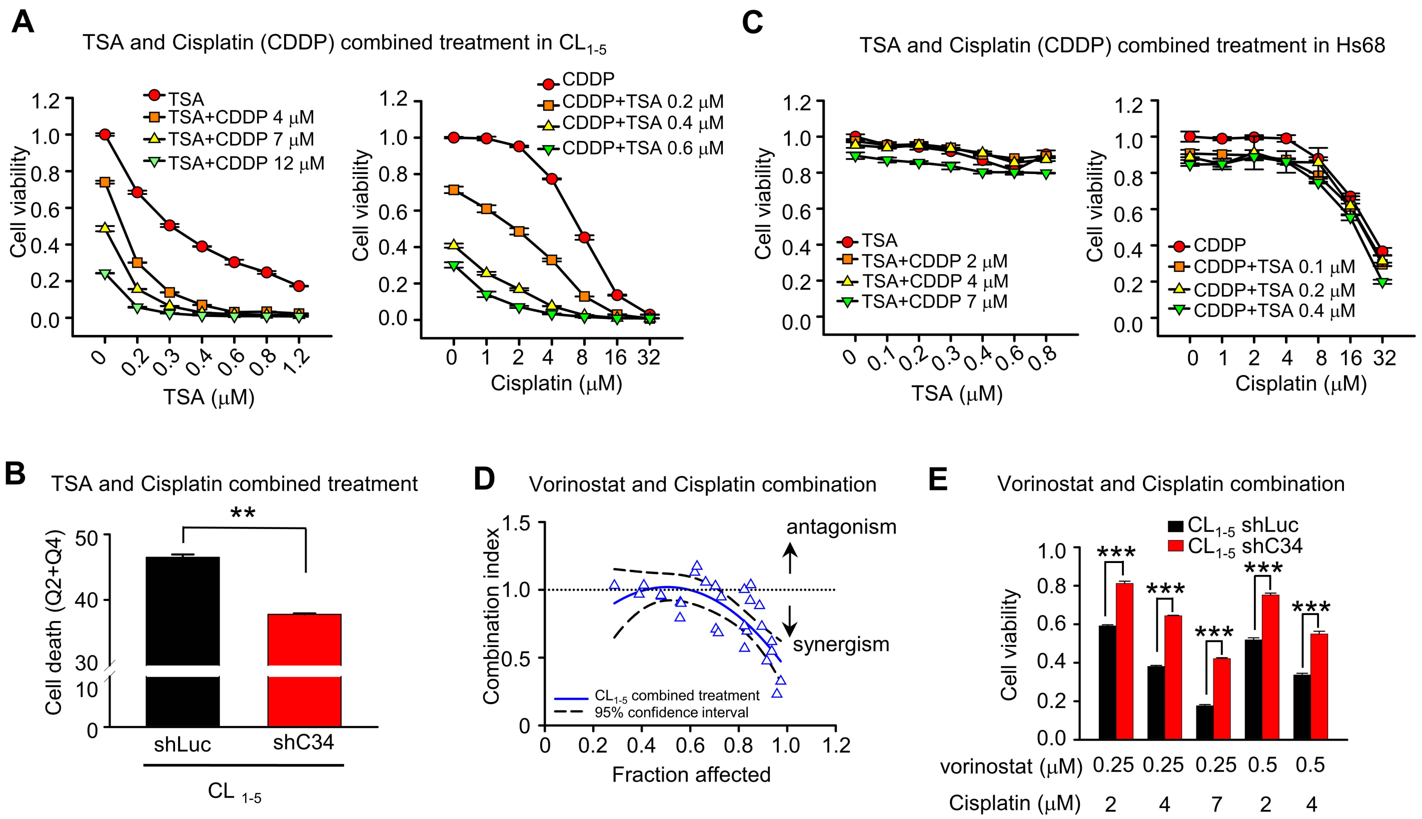

Supplementary Table S1. Antibodies

Proteins or secondary antibodies	Assay	Antibody	Origin	Dilution	Incubation period
CLDN1	WB	51-9000, Invitrogen	rabbit	1/1000	overnight, 4 °C
	WB	sc-166338, Santa Cruz Biotechnology, Inc	mouse	1/200	overnight, 4 °C
	IHC	51-9000, Invitrogen	rabbit	1/25	overnight, 4 °C
EPHB6	WB	sc-398795, Santa Cruz Biotechnology, Inc	mouse	1/400	overnight, 4 °C
	IHC	SAB1403784, Sigma-aldrich	mouse	1/500	overnight, 4 °C
RUNX3	WB, ChIP	MABE145, Merck Millipore	mouse	1/1000	overnight, 4 °C
SLUG	IHC	sc-166476, Santa Cruz Biotechnology, Inc	mouse	1/150	overnight, 4 °C
	WB, ChIP	sc10436, Santa Cruz Biotechnology, Inc	goat	1/1000	overnight, 4 °C
Snail	WB	MABE167, Merck Millipore	mouse	1/1000	overnight, 4 °C
Twist 1/2	WB	GTX127310, GeneTex	rabbit	1/1000	overnight, 4 °C
E-cad	WB	610182, BD	mouse	1/1000	overnight, 4 °C
p-ERK	WB	#9101, Cell Signaling Technology, Inc.	rabbit	1/50000	overnight, 4 °C
ERK	WB	#4695, Cell Signaling Technology, Inc.	rabbit	1/50000	overnight, 4 °C
p-JNK	WB	#4668, Cell Signaling Technology, Inc.	rabbit	1/1000	overnight, 4 °C
JNK	WB	#9258, Cell Signaling Technology, Inc.	rabbit	1/1000	overnight, 4 °C
p-p38	WB	#4511, Cell Signaling Technology, Inc.	rabbit	1/1000	overnight, 4 °C
p38	WB	#9212, Cell Signaling Technology, Inc.	rabbit	1/1000	overnight, 4 °C
caspase 3	WB	IMG-144A, Imgenex	mouse	1/1000	overnight, 4 °C
cleaved PARP	WB	#9546, Cell Signaling Technology, Inc.	mouse	1/1000	overnight, 4 °C
ACTIN	WB	A5441, Sigma-aldrich	mouse	1/200000	overnight, 4 °C
anti-myc tag	WB	05-419, Merck Millipore	mouse	1/5000	overnight, 4 °C
	IP	05-419, Merck Millipore	mouse	1 μg for 0.5 μg protein in 1000 μl	overnight, 4 °C
	ICC	05-419, Merck Millipore	mouse	1/500	overnight, 4 °C
anti-HA.11 tag	WB	MMS-101P, covance	mouse	1/5000	overnight, 4 °C
anti-Flag tag	WB	F1804, Sigma-aldrich	mouse	1/10000	overnight, 4 °C
Histone H3 (tri methtl K27), ChIP Grade	ChIP	ab6002. abcam	mouse	5 μg for 3 x 10^6 cells	overnight, 4 °C
Histone H3 (tri methyl K4), ChIP Grade	ChIP	ab1012, abcam	mouse	2 μg for 3 x 10^6 cells	overnight, 4 °C
Histone H3 (acetyl K9), ChIP Grade	ChIP	ab4441, abcam	rabbit	5 μg for 3 x 10^6 cells	overnight, 4 °C

Proteins or secondary antibodies	Assay	Antibody	Origin	Dilution	Incubation period
Histone H3 (acetyl K14), ChIP Grade	ChIP	ab52946, abcam	rabbit	5 μg for 3 x 10^6 cells	overnight, 4 °C
Histon H3 (tri methyl K9)	ChIP	07-442, Merck Millipore	rabbit	5 μg for 3 x 10^6 cells	overnight, 4 °C
normal mouse IgG	IP, IF, ChIP	12-371, Merck Millipore	mouse	the amount is equal to the test group	overnight, 4 °C
normal rabbit IgG	IP, ChIP	12-370, Merck Millipore	rabbit	the amount is equal to the test group	overnight, 4 °C
normal goat IgG	ChIP	sc-2028, Santa Cruz Biotechnology, Inc	goat	the amount is equal to the test group	overnight, 4 °C
Goat anti-mouse IgG-HRP	WB	115-035-003, Jackson ImmunoResearch	goat	1/5000	1 h, RT
Goat anti-rabbit IgG-HRP	WB	111-035-003, Jackson ImmunoResearch	goat	1/5000	1 h, RT
Bovine anti-goat IgG-HRP	WB	805-035-180, Jackson ImmunoResearch	Bovine	1/5000	1 h, RT
Goat anti-mouse DyLight 594	ICC/IF	ab96873, abcam	goat	1:200	1 h, RT
CD133	Flow	CD133/2 (293C3)-PE,130-090-853, Miltenyi Biotec	mouse	10 μl per 100 μl cell suspenssion (10^7 cells)	10 min, 4 °C

Supplementary Table S2. Sequences of the oligonucleotides

assay	gene		sequence 5'->3'
**shRNA**			
	shLuc		CAAATCACAGAATCGTCGTAT
	shLacZ		CGCGATCGTAATCACCCGAGT
	shCLDN1-33		CCACAGCATGGTATGGCAATA
	shCLDN1-34		CTGGGAGTGATAGCAATCTTT
	shEPHB6-51		GAGTGAGCAGGAGGTACTAAA
	shEPHB6-52		GAATGACGATACCCGTGACTC
	shRUNX3-674		GGCTAGCAGCATGCGGTATTT
	shRUNX3-675		ACCACCTCTACTACGGGACAT
	shSLUG-S1		GAACTGGACACACATACAGTG
	shSLUG-S2		GAGGAAAGACTACAGTCCAAG
	shSLUG-S3		CCCATTCTGATGTAAAGAAAT
**bisulfite sequencing primers**			
	CLDN1 promoter	forward	TATTAAATTTAAAATTGTAGTTTTTGAAGG
		reverse	TCACACAACAAAATAAACC CATAAA
**Methylation-specific PCR**			
The methylated primers	CLDN1 promoter	forward	TTCGTTTTAATTTTTTTCGCGGGGTTT
		reverse	CGATAACGCCGATCCATCCC
The unmethylated primers	CLDN1 promoter	forward	TTTTGTTTTAATTTTTTTTGTGGGGTTTA
		reverse	CTAACAATAACACCAATCCATCCCA
For bisulfite converted sequence	β-ACTIN	forward	TGGTGATGGAGGAGGTTTAGTAAGT
		reverse	AACCAATAAAACCTACTCCTCCCTTAA
**pyrosequencing of CpG percentage**			
amplicon1	CLDN1 promoter	forward	GGGAGTAATAGTAGTTTTTAGTATTTAGAT
		reverse	biotin-ATAAAACCCAACAACTACAACC
		sequencing	GATTTTAATTTAGATTTAGAGTTTT
amplicon2	CLDN1 promoter	forward	GGGAGTAATAGTAGTTTTTAGTATTTAGAT
		reverse	biotin-ATAAAACCCAACAACTACAACC
		sequencing	AATTTTTTTAGAGGGGTTTAGTTAT
amplicon3	CLDN1 promoter	forward	GGGAGTTAGGGTTGTTTATTTGTAAA
		reverse	biotin-ATAAAACCCAACAACTACAACC
		sequencing	GTTTTTTGTATTTGTTATTTTTGA
**RT-qPCR**			
	CLDN1	forward	CCGTTGGCATGAAGTGTATG
		reverse	AGCCAGACCTGCAAGAAGAA
	EPHB6	forward	ATGATCCGCAAGCCAGATAC-
		reverse	GGGTGAGTCCAGACAAGGAA
	RUNX3	forward	AGTGGGCGAGGGAAGAGTT
		reverse	AGTGGCTTGTGGTGCTGAGT
	SLUG	forward	ACAGCGAACTGGACACACATAC
		reverse	TCTCTGGTTGTGGTATGACAGG
	SNAIL	forward	ACCACTATGCCGCGCTCT
		reverse	GGTCGTAGGGCTGCTGGAA
	ZEB1	forward	TTCAAACCCATAGTGGTTGCT
		reverse	TGGGAGACACCAAACCAACTG
	ALDH1A1	forward	TGAATTGCTATGGCGTGGTA
		reverse	GGAAACCGTACTCTCCCAGTT
	NANOG	forward	AATACCTCAGCCTCCAGCAGATG
		reverse	TGCGTCACACCATTGCTATTCTTC
	NES	forward	CAGCTGGCGCACCTCAAGATG
		reverse	AGGGAAGTTGGGCTCAGGACTGG
	OCT4	forward	ACATCAAAGCTCTGCAGAAAGAACT
		reverse	CTGAATACCTTCCCAAATAGAACCC
	GAPDH	forward	TGAAGGTCGGAGTCAACGGATT
		reverse	CCTGGAAGATGGTGATGGGATT
**ChIP**			
ChIP primer1	CLDN1 promoter	forward	CTCCCCGCCTTAACTTCCT
		reverse	AGGAAGGCGAGAATGAAGC
ChIP primer2	CLDN1 promoter	forward	TTGGATAATTGGAGTGAATGAATG
		reverse	CAGGACCAGGCACCAGAG
ChIP primer3	CLDN1 promoter	forward	CCTTTCCTTCTCTGTCACCAA
		reverse	TTTTTGTGTGTGGTGCGAGT
